# Horizontally acquired antibacterial genes associated with adaptive radiation of ladybird beetles

**DOI:** 10.1186/s12915-020-00945-7

**Published:** 2021-01-14

**Authors:** Hao-Sen Li, Xue-Fei Tang, Yu-Hao Huang, Ze-Yu Xu, Mei-Lan Chen, Xue-Yong Du, Bo-Yuan Qiu, Pei-Tao Chen, Wei Zhang, Adam Ślipiński, Hermes E. Escalona, Robert M. Waterhouse, Andreas Zwick, Hong Pang

**Affiliations:** 1 State Key Laboratory of Biocontrol, School of Life Sciences / School of Ecology, Sun Yat-sen University, Guangzhou, 510275 China; 2 School of Environment and Life Science, Nanning Normal University, Nanning, 530001 China; 3 Australian National Insect Collection, CSIRO, GPO Box 1700, Canberra, ACT 2601 Australia; 4 Department of Ecology and Evolution, University of Lausanne and Swiss Institute of Bioinformatics, 1015 Lausanne, Switzerland

**Keywords:** Horizontal gene transfer, Antibacterial activity, Cell wall hydrolase, Ladybirds

## Abstract

**Background:**

Horizontal gene transfer (HGT) has been documented in many herbivorous insects, conferring the ability to digest plant material and promoting their remarkable ecological diversification. Previous reports suggest HGT of antibacterial enzymes may have contributed to the insect immune response and limit bacterial growth. Carnivorous insects also display many evolutionary successful lineages, but in contrast to the plant feeders, the potential role of HGTs has been less well-studied.

**Results:**

Using genomic and transcriptomic data from 38 species of ladybird beetles, we identified a set of bacterial *cell wall hydrolase* (*cwh*) genes acquired by this group of beetles. Infection with *Bacillus subtilis* led to upregulated expression of these ladybird *cwh* genes, and their recombinantly produced proteins limited bacterial proliferation. Moreover, RNAi-mediated *cwh* knockdown led to downregulation of other antibacterial genes, indicating a role in antibacterial immune defense. *cwh* genes are rare in eukaryotes, but have been maintained in all tested Coccinellinae species, suggesting that this putative immune-related HGT event played a role in the evolution of this speciose subfamily of predominant predatory ladybirds.

**Conclusion:**

Our work demonstrates that, in a manner analogous to HGT-facilitated plant feeding, enhanced immunity through HGT might have played a key role in the prey adaptation and niche expansion that promoted the diversification of carnivorous beetle lineages. We believe that this represents the first example of immune-related HGT in carnivorous insects with an association with a subsequent successful species radiation.

**Supplementary Information:**

The online version contains supplementary material available at 10.1186/s12915-020-00945-7.

## Background

Acquisitions by organisms of genetic material from other species, i.e., horizontal gene transfers (HGTs) or lateral gene transfers, play a key role in the evolution of both prokaryotes (Bacteria and Archaea) and eukaryotes [1, 2]. HGTs occur frequently among prokaryotes, but prokaryote to eukaryote gene transfers are far less prevalent [3, 4]. Wider genomic sampling of animals is nevertheless revealing many such cases of HGT [2, 5], although the extent is debated [6, 7] and some claims may be confounded by contamination [8]. Growing evidence indicates that hosts gain important functional innovations from these HGTs that can confer powerful adaptive advantages, such as expanded feeding capacities or new defenses to protect themselves from other organisms [2, 9, 10].

Long-standing intimate relationships between insects and microbes make them good candidates for HGT detection [11]. Prominent examples include HGTs that have contributed to the evolution of herbivory in arthropods and nematodes by facilitating plant cell wall digestion and metabolite assimilation and overcoming plant defenses [12, 13]. Plant cell wall-degrading enzymes acquired from bacteria and fungi have been identified in many insect species [14] and are considered to have been vital to the evolutionary success of beetles [15–19]. Other HGTs protect mites, moths, and butterflies from plant-produced cyanide [20] or enable sap-feeding aphids to synthesize their own carotenoids [21]. Acquisitions not directly linked to plant feeding appear to be rarer and include HGTs that enhance eukaryotic innate immune defenses [22]. For example, deer ticks have genes encoding prokaryotic type VI secretion amidases that degrade bacterial cell walls [23], peptidoglycan-degrading bacterial lysozymes have been detected in insects [24, 25] and nematodes [5], and fruit flies have genes related to phagocytosis of bacteria [5].

We report a putative immune-related HGT event from bacteria to eukaryotes. A bacterial cell wall hydrolase (*cwh*) gene was maintained throughout the radiation of a large subfamily of diverse ladybird beetles, while noticeably absent from most other insect genomes screened. These *cwh* genes are expressed in different tissues and life stages and are upregulated on infection with Gram-positive bacteria, and recombinantly expressed *cwh* proteins limit bacterial proliferation. Moreover, suppression of *cwh* expression through RNA interference (RNAi) led to downregulation of other antibacterial genes. These ladybirds exhibit a broad range of diets but are overwhelmingly predators of aphids, coccids, psyllids, and whiteflies, which usually host a wide range of bacterial symbionts [26]. We conclude that, in a manner analogous to HGT-facilitated plant feeding, enhanced immunity through HGT could have played a role in prey adaptation and niche expansion that promoted the diversification of carnivorous beetle lineages.

## Results and discussion

### Ladybird *cwh* genes encode bacterial cell wall hydrolases

**Fig. 1:**
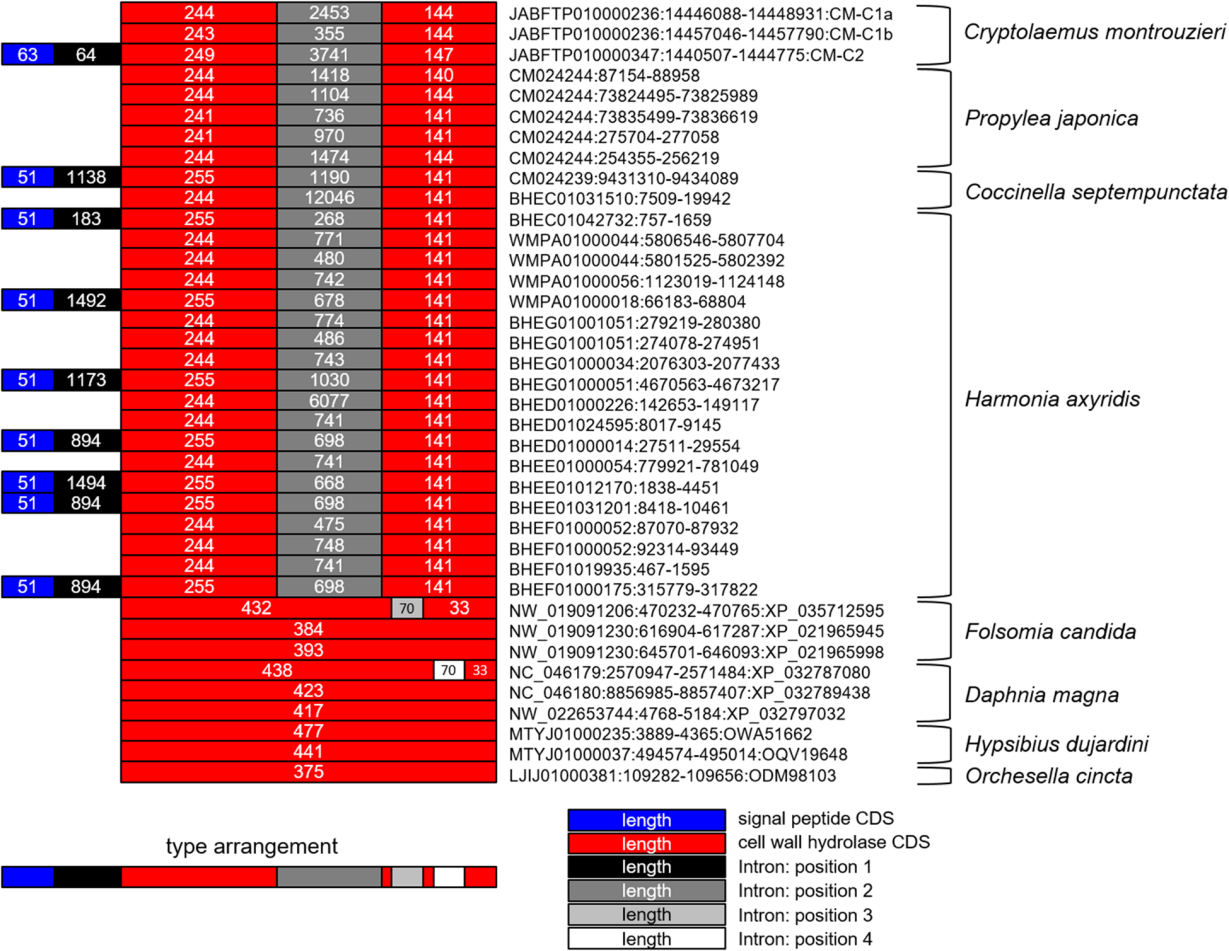
Genomic features of putative eukaryotic *cwh* genes. Genomes of four ladybird species and high-quality genomes of four other eukaryotic species were studied

We detected a set of *cell wall hydrolase* (*cwh*) genes containing the domain of “Hydrolase_2” (Pfam accession: PF07486) from the high-quality genome of a ladybird species *Cryptolaemus montrouzieri* (NCBI BioProject: PRJNA626074). These genes, without and with a signal peptide, were named *cwh1* and *cwh2*, respectively. Homologs of both *cwh1* and *cwh2* were detected from all published genomes of Coccinellinae species, i.e., *Propylea japonica* [27], *Coccinella septempunctata*, and *Harmonia axyridis* [28, 29] (Fig. 1). These genes encode bacterial cell wall hydrolases but are not derived from contaminated bacterial DNA. This is evidenced by their locations in the beetle genomes, they are flanked by eukaryotic genes (Additional file 1: Fig. S1). Furthermore, they contain introns (Fig. 1), which are absent from genes in bacteria, they share the same splice sites, and they have Guanine-Cytosine contents (GC%) that are similar to the flanking sequences (Additional file 1: Fig. S2). The function of *cwh* genes has been characterized in *Bacillus* as encoding soluble lytic transglycosylases that specifically recognize spore cortex peptidoglycan and catalyze the cleavage of glycosidic bonds between N-acetylmuramic acid (NAM) and N-acetylglucosamine residues, with concomitant formation of a 1,6-anhydro bond in the NAM residue [30, 31].

We next explored the regular expression of ladybird *cwh* genes in *C. montrouzieri*, *H. axyridis*, and *P. japonica* for different spatial and temporal patterns. There was no significant spatial differentiation of *cwh* expression among male head-thorax, gut, and gut-removed abdomen tissues (Additional file 1: Fig. S3). Additionally, the expression levels of *cwh* were generally similar for the 4th instar larva, pupa, and male adult stages (Additional file 1: Fig. S4). This finding indicates that *cwh* genes are functionally integrated into the physiology of ladybirds across the whole body and probably the entire life cycle.

**Fig. 2:**
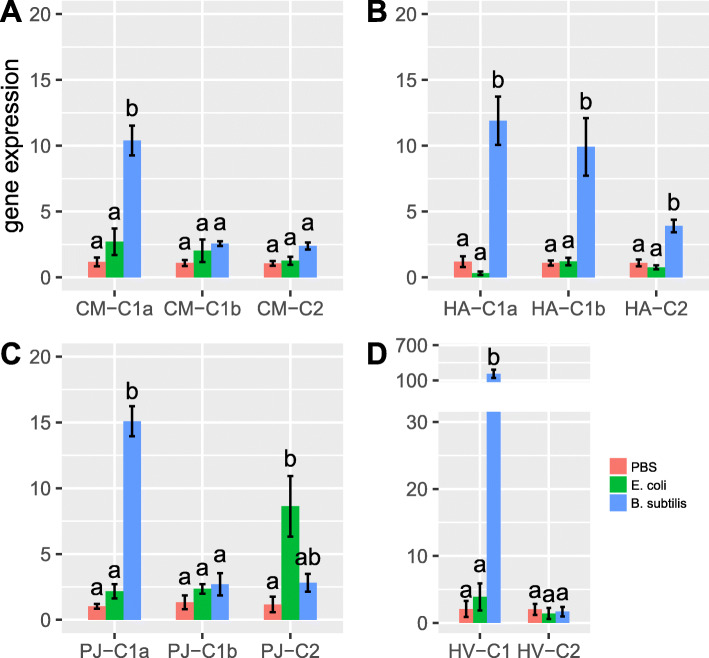
Evidence of the antibacterial activity of ladybird *cwh* genes I: Upregulation of *cwh* in response to bacterial infection. The expression patterns of *cwh* genes in response to bacterial infection (*E. coli* and *B. subtilis*) were tested in **a** *Cryptolaemus montrouzieri.* (CM-C1a, CM-C1b and CM-C2), **b** *Harmonia axyridis* (HA-C1a, HA-C1b and HA-C2), **c** *Propylea japonica* (PJ-C1a, PJ-C1b and PJ-C2), and **d** *Henosepilachna vigintioctopunctata* (HV-C1 and HV-C2). PBS treatments were used as control. Expression levels of *cwh* were normalized to those of two reference genes (only one of them is shown; see details of two reference genes in Additional file 1: Table S2 and the comparative results in Additional file 1: Fig. S5). Relative expression of each *cwh* gene in comparison to the average level of that gene in PBS treatment was analyzed by the 2^−ΔΔCt^ method to calculate the fold changes. Error bars show ± standard errors with biological replicates. Bars with the same letter are not significantly different (*p* ≥ 0.05)

**Fig. 3:**
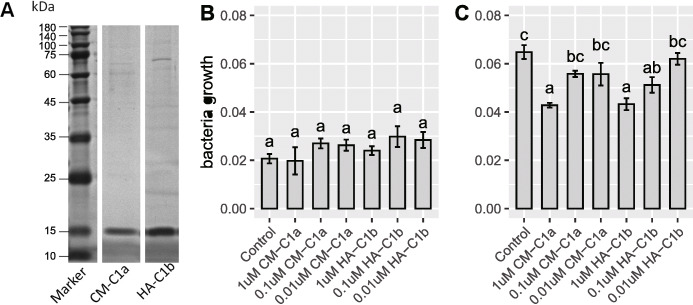
Evidence of the antibacterial activity of ladybird *cwh* genes II: Recombinant protein activity that limited bacterial proliferation. **a** Western blot of the recombinant proteins of *cwh1* suggested the success of cloning and recombinant expression. The bacterial killing activities of *cwh1* proteins against **b** *Escherichia coli* and **c** *Bacillus subtilis* cells were measured by the increase in OD_630 nm_ within 90 min of each treatment. Error bars show ± standard errors with biological replicates. Bars with the same letter are not significantly different (*p* ≥ 0.05)

**Fig. 4:**
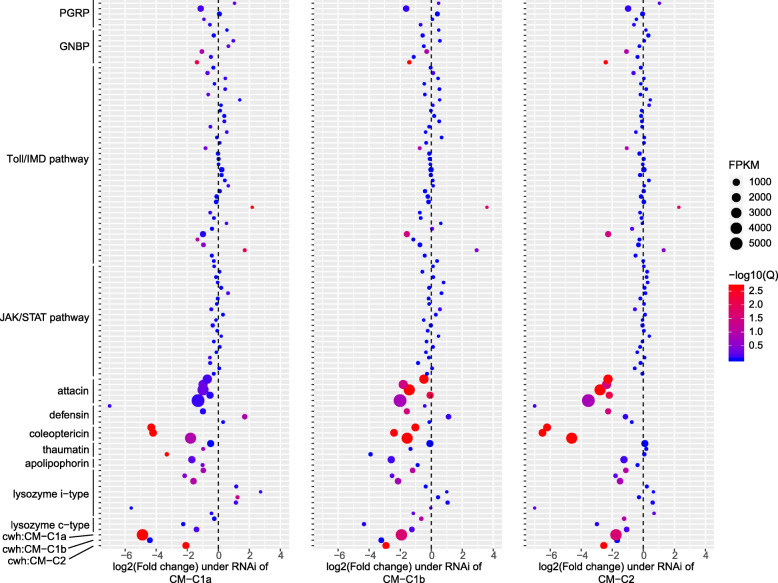
Evidence of the antibacterial activity of ladybird *cwh* genes III: Downregulation of other antibacterial genes in *cwh*-RNAi strains. Gene expression levels of three *cwh*-RNAi strains of *Cryptolaemus montrouzieri* (RNAi of CM-C1a, CM-C1b, and CM-C2) were studied based on transcriptome sequencing data. Three biological replicates were set for each treatment, and GFP-treated individuals were used as control. Patterns of expression of immunity-related genes involving in recognition (PGRP and GNBP), signal transduction (Toll/IMD and JAK/STAT pathway), and defense execution (antimicrobial peptides (AMPs) and lysozymes) are shown. FPKM (fragments per kilobase of transcript per million fragments mapped) of PBS controls, log2(fold change) and -log10 adjust-*p* values (-log10(Q)) for the transcriptome comparisons of *cwh*-RNAi strains vs PBS control were showed. Only genes with average FPKM higher than 1 are shown

We validated the functional relevance of ladybird *cwh* genes through three strategies. Firstly, the ladybird species *C. montrouzieri*, *H. axyridis*, *P. japonica*, and *Henosepilachna vigintioctopunctata* (with *cwh* genes identified from the transcriptome) were artificially infected with *Escherichia coli* (Gram-negative bacteria) and *Bacillus subtilis* (Gram-positive bacteria) through injection. The expression of *cwh* genes in the infected ladybirds was measured after 24 h through quantitative PCR (qPCR). In all four ladybird species, *cwh1* (C1a and C1b), unlike *cwh2* (C2)*,* was significantly upregulated following infection with *B. subtilis* but not with *E. coli* (Fig. 2 and details in Additional file 1: Fig. S5). Specifically, we detected extreme upregulation of *cwh1* in a plant-feeding ladybird following infection (Fig. 2d). Secondly, the effect of recombinantly expressed *cwh1* proteins from two ladybird species (CM-C1a of *C. montrouzieri* and HA-C1b of *H. axyridis*) on bacterial growth was tested. Both recombinant *cwh1* proteins limited the proliferation of *B. subtilis* significantly but did not limit *E. coli* proliferation (Fig. 3). Thirdly, the transcriptome response of RNA interference (RNAi)-mediated *cwh* knockdown was studied. All three *cwh*-RNAi strains of *C. montrouzieri* (RNAi of CM-C1a, CM-C1b, and CM-C2) showed downregulation of most genes encoding antimicrobial peptides (AMPs) and lysozymes but few other immunity-related genes (Fig. 4). These expression patterns suggest that *cwh* genes do not play roles in immune recognition or signaling processes but instead act together with AMPs and lysozymes, possibly in a synergistic manner, to execute immune defense responses. Taken together, the results of these three tests strongly support antibacterial activity against Gram-positive bacteria for *cwh* alone or synergistic action of *cwh* and other antibacterial genes, which could form part of the immune effector response of ladybirds.

### Ladybird *cwh* genes horizontally transferred from bacteria

**Fig. 5:**
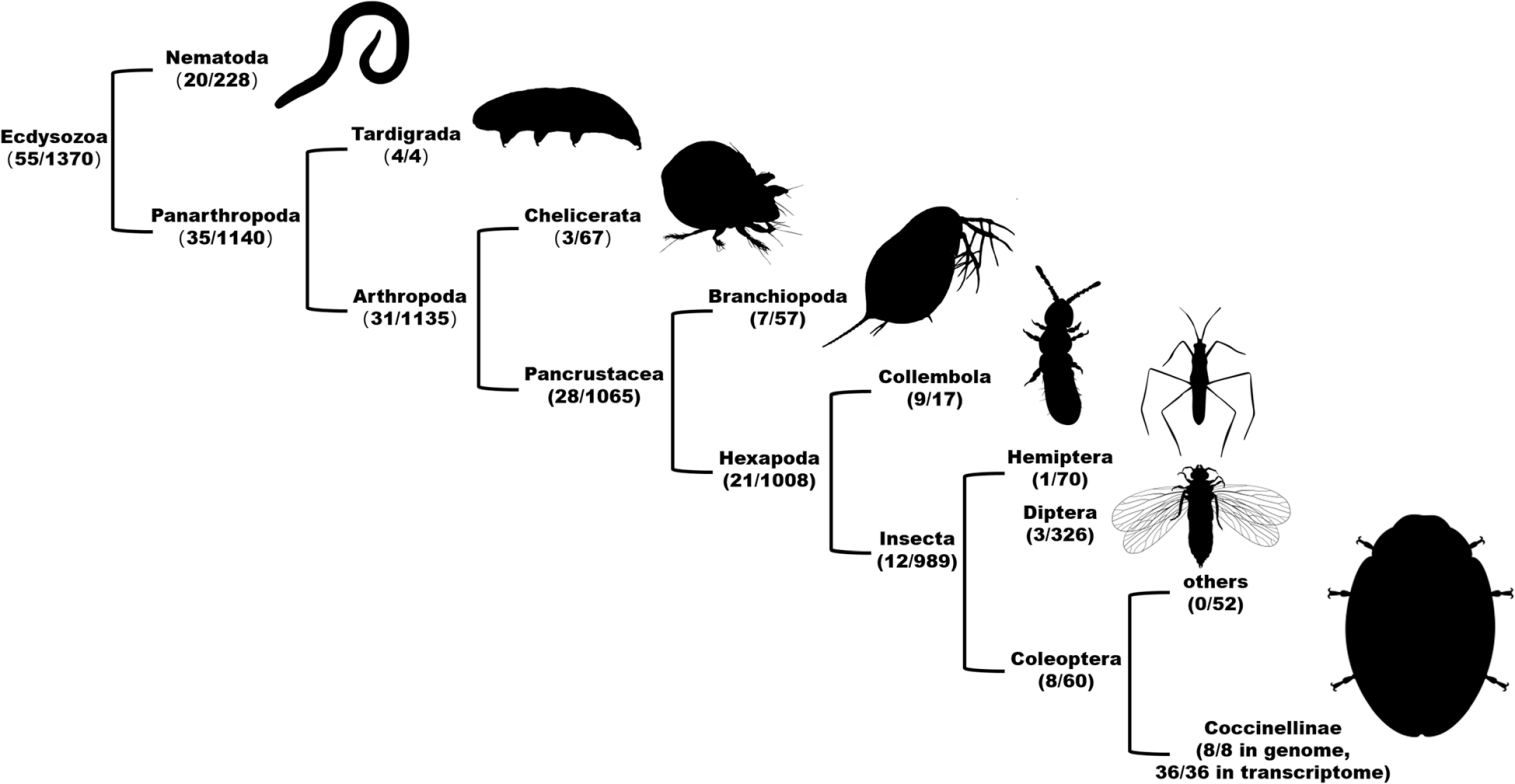
Occurrence of putative *cwh* genes in edcysozoan genomes deposited in NCBI whole genome shotgun (WGS) assembly database. Taxon pictures are courtesy of PhyloPic (http://www.phylopic.org/)

**Fig. 6:**
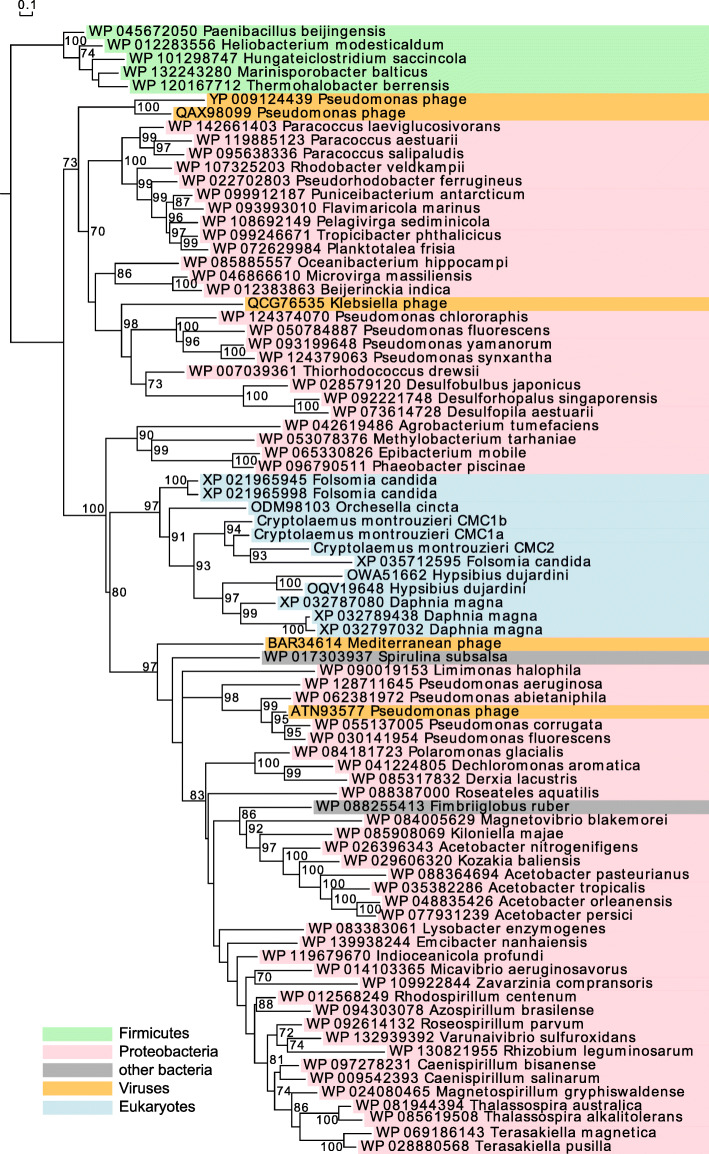
Evidence of horizontal gene transfer. The phylogenetic trees of representative *cwh* protein sequences were reconstructed in a maximum likelihood framework. The tree was rooted by the clade comprising mainly Firmicutes. Only node support values > 70% are shown. The NCBI protein sequence IDs and species names are color-coded according to the taxonomic group of the organisms

In addition to the ladybirds we examined, *cwh* homologs were also detected in four high-quality gene sets in eukaryotic genomes, i.e., the springtails *Folsomia candida* [32] and *Orchesella cincta* [33], the water flea *Daphnia magna* [34], and the water bear *Hypsibius dujardini* [35]. These putative *cwh* genes from the genomes of ladybirds, springtails, water fleas, and water bears are flanked by eukaryotic genes (Additional file 1: Fig. S1) and unlikely to be derived from contaminating bacterial DNA. Contrary to the ladybird *cwh*, all of these non-ladybird homologs lack the signal peptide, have different splice sites from each other and the ladybirds, or have no intron at all (Fig. 1). A broader BLAST search of genome assemblies in NCBI suggests that *cwh* homologs are potentially present in only 55 ecdysozoan genomes (Fig. 5; taxonomic details in Additional file 1: Table S4). These putative eukaryotic *cwh* genes are generally rare in genomes of Ecdysozoa (55/1370) but maintained in all tested Coccinellinae (8/8). Phylogenetic analysis of the putative eukaryotic and representative bacterial sequences of *cwh* proteins shows that the ladybird *cwh* genes and the other putative eukaryotic *cwh* genes form a monophyletic group (ultrafast bootstrap, UFBoot 97%) (Fig. 6). This eukaryotic *cwh* group is embedded within a large group of mainly Proteobacteria (UFBoot 100%), indicative of a bacterium-to-eukaryote HGT. The monophyly of this eukaryotic *cwh* group can be explained by (1) a single HGT event in the ecdysozoan ancestor, followed by vertical inheritance and independent loss in multiple lineages (a similar explanation in Chou et al. [23]), or (2) a small number of independent HGT events could have given rise to the observed groupings if donors were from lineages of Proteobacteria that have not been sequenced (a similar suggestion in Wybouw et al. [20]), or (3) a bacterium-to-eukaryote transfer, followed by multiple eukaryote-to-eukaryote transfers. A single ancestral acquisition of a *cwh* gene with subsequent divergence through speciation of the hosts (scenario #1) should result in a gene tree that follows the ecdysozoan phylogeny. However, the branching pattern within this eukaryotic *cwh* group does not match the host species phylogeny (Fig. 6; more extensive taxon sampling in Additional file 1: Fig. S6), which probably reflects too short and rapidly evolving gene sequences for deep phylogenetic reconstruction, but would also be congruent with the occurrence of multiple HGTs (scenario #2; [36]).

### Potential role of *cwh* genes in diversification of ladybirds

Of the examined 38 sequenced transcriptomes of ladybird species (family Coccinellidae) (Additional file 1: Table S3), the 36 species of the subfamily Coccinellinae included *cwh* genes, whereas the two species of the subfamily Microweiseinae lacked *cwh* genes completely. Since most of the Coccinellinae species have both *cwh1* and *cwh2* genes, while none of the *cwh* genes is present in Microweiseinae and other Coleoptera outgroups, we inferred that *cwh* genes were present in the common ancestor of Coccinellinae in the Cretaceous, and maintained throughout their radiation.

**Fig. 7:**
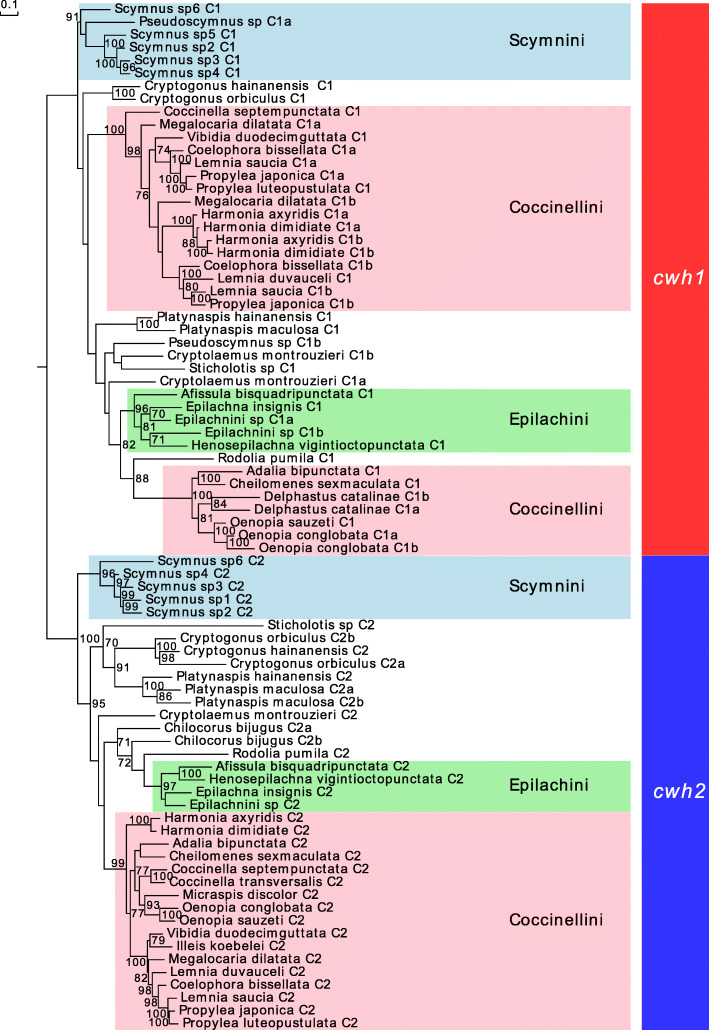
Evolutionary history of ladybird *cwh* genes. The phylogenetic tree of *cwh* protein sequences of ladybirds was reconstructed in a maximum likelihood framework. The ladybird *cwh* genes are separated into two clades, *cwh1* (suffix C1 or C1a or C1b) and *cwh2* (suffix C2 or C2a or C2b)

A phylogenetic analysis based on the sequences from the ladybird *cwh* protein sequences alone suggests that both the *cwh1* and *cwh2* of ladybirds are monophyletic (UFBoot 100%) (Fig. 7). The topologies within *cwh1* and *cwh2* gene trees support the monophyly of the ladybird tribes Scymnini (UFBoot 91%) and Epilachini (UFBoot 96%) for *cwh1*, and of Scymnini (UFBoot 96%), Epilachini (UFBoot 97%), and Coccinellini (UFBoot 99%) for *cwh2* (Fig. 7). The consistent retention of either *cwh1* or *cwh2* by all 36 Coccinellinae species and the duplications of the gene in some lineages (e.g., two *cwh1* in Coccinellini) provide evidence of *cwh*’s indispensable function. We also found that the glutamate catalytic domain that is present in most of the bacterial *cwh* enzymes [37, 38] is also present in the ladybird data analyzed (Additional file 4), suggesting the retention of bacterial cell wall hydrolase activity and the ability to act as a toxin against bacteria. In addition, major signals of negative selection act within both *cwh1* and *cwh2* (Additional file 1: Table S5), indicating also that these genes are functionally conserved.

The two *cwh* genes in ladybirds might have different functions. Their distribution in the phylogenetic tree (Fig. 7) indicates that the two clades *cwh1* and *cwh2* probably diverged before the diversification of Coccinellinae ladybirds into the extant species. Moreover, *cwh2* contains a signal peptide in the N-terminus, while *cwh1* does not (Fig. 1). This suggests that *cwh2* is unique in protein assembly or export from eukaryotic cells. Both *cwh1* and *cwh2* subsequently underwent independent gene duplications in some taxa (resulting in C1a, C1b, C2a, and C2b). The *cwh* gene family phylogeny and the position of these genes in the genome suggest that, at least in some taxa, these duplications occurred recently. For example, the *cwh1* genes of *Harmonia* species are closely related (Fig. 7), and the two *cwh1* genes of *C. montrouzieri* are separated by a gap of only ~ 8000 bp. In addition, the duplications of *cwh1* occurred mainly in Coccinellini (Fig. 7), which is a group with particularly mixed diets [39]. This suggests that the function of *cwh1* might be essential for diet expansion.

**Fig. 8:**
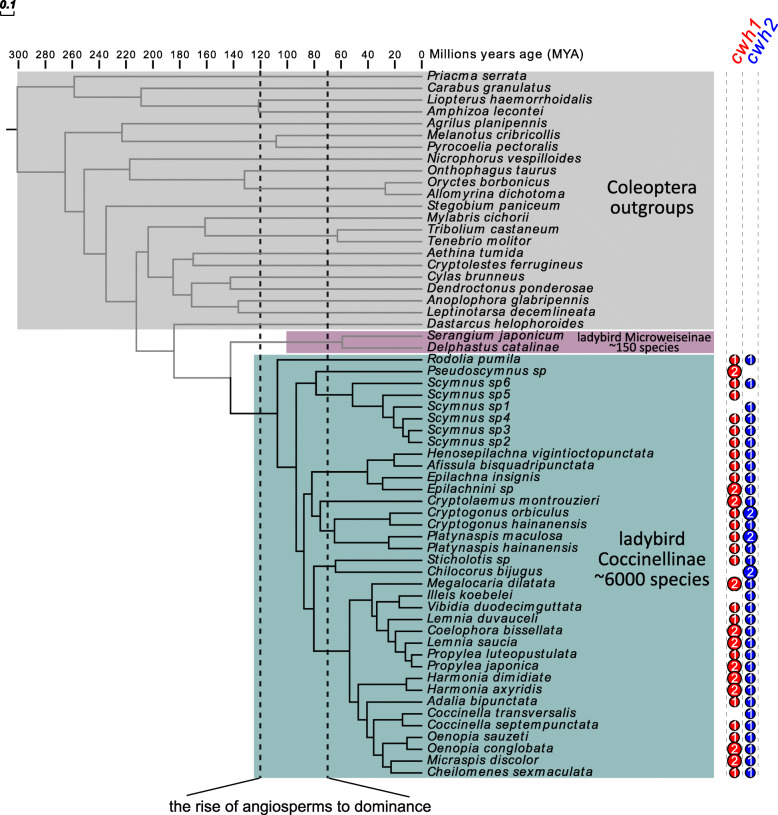
The consistent retention of *cwh* genes from the beginning of angiosperm dominance is considered relevant to the adaptive radiation of ladybirds. A phylogenetic study of ladybird Coccinellidae and Coleoptera outgroups based on 819 single-copy genes and 12 fossil calibration points revealed crown diversification of the ladybird subfamily Coccinellinae approximately 110 million years ago (MYA). Most of the Coccinellinae species contain both *cwh1* (red disc) and *cwh2* (blue disc). Details of the tree can be found in Additional file 1: Figs. S7 and S8

Based on phylogenomic and molecular clock analyses of transcriptomes (819 single-copy genes) in 60 beetles, including 38 ladybird species (Additional file 1: Table S3), we estimate the divergence of major ladybird lineages to have occurred in the Cretaceous, approximately 110 million years ago (MYA) (108.8 MYA, 95% confidence interval: 134.1–83.0 MYA) (Fig. 8 and details of phylogenetic trees in Additional file 1: Figs. S7 and S8), concurrent with the rise of angiosperms to widespread floristic dominance [40]. The extraordinary diversity of herbivorous beetles has been attributed to a Cretaceous angiosperm explosion [41]. However, some insects (e.g., weevil beetles, butterflies) did not co-diversify with angiosperms but had shorter or longer temporal lags instead [42, 43]. This lag is believed to have been caused by the dependence of their evolution on not only the diversification of resources and ecological opportunities but also the ability of these taxa to utilize them [44]. The latter might be the case for ladybird beetles, too. Unlike ladybird beetles, most of the related families in the superfamily Coccinelloidea are fungus feeders, which suggests that ancestral ladybirds shifted their diet from fungi to prey [41, 45, 46]. In the Cretaceous, a large ecological opportunity arose for ladybirds with the diversification of angiosperms and their herbivorous Sternorrhyncha communities (coccids, aphids, whiteflies, and psyllids) [47, 48]. The consistent retention of antibacterial functions through *cwh* genes in the subfamily Coccinellinae might have been an advantage for prey adaptation in ladybirds. A genome-wide scan of gene family evolution of all the published Coleoptera genomes showed that Coccinellinae genomes (including *C. montrouzieri*, *H. axyridis*, and *C. septempunctata*) had more AMP and lysozyme genes, which have a similar antibacterial function to that of *cwh*, than most of the other beetles (Additional file 1: Fig. S9) [18, 19, 28, 49–55]. Further, insect antimicrobial properties, including AMPs and lysozymes, usually have synergistic actions [56–58]. *cwh* proteins and lysozymes both target bacterial peptidoglycans but act on different sites, suggesting their specificity of antibacterial activity. While ladybirds have quite a large number of antibacterial genes encoding AMPs such as attacins, defensins, thaumatins, and coleoptericins and lysozymes [59–61], *cwh* products might provide a broader antibacterial spectrum or enhance antibacterial efficiency of other antimicrobials [62]. Besides ladybirds, *cwh* genes are mainly known from aquatic and soil-dwelling eukaryotes including nematodes, water bears, oribatid mites, water fleas, and springtails. Several other HGT events have been reported for these groups, which might contribute to adaptation to habitats with diverse microbial communities [32, 33, 63]. Similarly, it is possible that ladybirds maintain *cwh* genes that enable them to thrive on a wide range of diets, including the prevalent diet of Sternorrhyncha, which is known to contain a particularly large diversity of bacterial commensals [26].

## Conclusion

We identified a set of eukaryotic *cwh* genes that encode bacterial cell wall hydrolases. These genes probably originated from bacteria and maintained in all tested ladybird species of the subfamily Coccinellinae. Experimental evidence demonstrates antibacterial activity by at least one of two *cwh* gene products. We therefore suggest that HGT might be key in the acquisition of immune system enhancements, which in turn promotes the adaptation and radiation of ladybirds during the rise of angiosperms and their sternorrhynchan herbivores during the Cretaceous.

## Materials and methods

### Detection of *cwh* genes and the other immune-related genes in ladybird genomes

The genome sequences of the ladybird *C. montrouzieri* are from our unpublished data (NCBI BioProject: PRJNA626074). The predicted genes from this genome were annotated with the Pfam v32.0 database (http://pfam.xfam.org) using InterProScan v5.35 [64]. The Hydrolase_2 (Pfam accession: PF07486) domain-containing genes were identified as *cwh* genes. The presence of introns and flanking eukaryotic sequences are considered evidence of a eukaryotic *cwh* gene. The GC% (100 bp sliding window) values of the *cwh* genes and their flanking sequences were investigated. The presence of signal peptides in the N-terminus of ladybird *cwh* genes was predicted by SignalP 5.0 [65] using the default cut-off. *cwh* genes were also detected in the other ladybird genomes deposited in NCBI using tBLASTn with a cut-off e-value of 10^− 10^.

In addition, other immunity-related genes of the published ladybird and other Coleoptera genomes were identified based on the annotation of Pfam, SwissProt, and KEGG pathways, including those encoding peptidoglycan recognition protein (PGRP, SwissProt), Gram-negative binding protein (GNBP, SwissProt), Toll and IMD pathway (KEGG: map 04624), JAK/STAT pathway (KEGG: map 04630), attacin (PF03769: Attacin, C-terminal region), defensin (PF01097: Arthropod defensin), coleoptericin (PF06286: Coleoptericin), thaumatin (PF00314: Thaumatin family), apolipophorin (SwissProt), and c-type and i-type lysozymes (SwissProt and Pfam).

### Spatial and temporal expression of *cwh* genes and expression during bacterial infection

The expression patterns of *cwh* genes of *C. montrouzieri*, *H. axyridis*, and *P. japonica* in different life stages and tissues were explored. The level of expression in 4th instar larva, pupa, and male adult stages, as well as in the head, gut, and gut-removed abdomen of male adults, was quantified by quantitative PCR (qPCR).

The expression patterns of *cwh* genes in response to infection by *E. coli* and *B. subtilis* were explored in *C. montrouzieri*, *H. axyridis*, *P. japonica*, and *H. vigintioctopunctata*. The bacterial cells were suspended at approximately 10^9^ CFU/mL using phosphate-buffered saline (PBS). We injected 2 mL of the suspension into the species with small body sizes (*C. montrouzieri* and *P. japonica*) and 5 mL of the suspension into the species with large body sizes (*H. axyridis* and *H. vigintioctopunctata*) in the abdomens of male adults. Insects injected with the same volume of PBS were set as controls. Total RNA was extracted 24 h after infection for further qPCR.

The primers for *cwh* and the reference genes (Additional file 1: Table S2) were designed using NCBI primer-BLAST based on the transcriptome data (see the following methods for transcriptome sequencing) or referenced from previous studies [66–68]. qPCR was performed on cDNA samples using a SYBR Green-based kit (Invitrogen, USA) according to the manufacturer’s protocol. Expression levels for *cwh* were normalized to those of two reference genes. Analyses of *cwh* expression included four technical replicates for each biological replicate and five biological replicates for each treatment. The Ct data of qPCR can be found in Additional file 2. Relative gene expression of each *cwh* in comparison to the average level of that in head and larva was analyzed by the 2^−ΔΔCt^ method to calculate the fold changes [69]. To test for significant differences in the expression levels of each *cwh* gene under different temporal, spatial, and bacterium-infected conditions, we applied one-way ANOVAs followed by Tukey’s post hoc tests using SPSS 17.0 (SPSS Inc).

### Tests of antibacterial activities of recombinant *cwh1* proteins

Since we found that ladybird *cwh1* genes were upregulated under bacterial infection in the above experiments, we further tested the effect of recombinant *cwh1* protein on bacterial growth. Two *cwh1* genes from *C. montrouzieri* and *H. axyridis* (CM-C1a and HA-C1b) were isolated by PCR from cDNA. The PCR primers were designed (Additional file 1: Table S6) to amplify the whole coding sequence. The resulting PCR products were originally cloned into the pEASY-Blunt E1 vector (TransGen, Beijing). The resulting construct was transformed into *E. coli* bacterial strain BL21 cells. The transformed cells were grown at 37 °C in LB medium containing 100 mg/mL ampicillin. When the bacterial culture reached an optical density of 0.5 at 600 nm (OD_600 nm_), protein synthesis was induced by the addition of 0.5 mM isopropyl 1-thio-b-galactopyranoside (IPTG). Four hours after induction, the cells were harvested by centrifugation at 8000×*g* for 10 min at 4 °C. After harvesting, the bacterial cells were resuspended and sonicated in buffer A (300 mM NaCl, 50 mM NaH_2_PO_4_, 10 mM imidazole, 10 mM Tris base, pH 8.0). After sonication, the cell suspension was centrifuged at 20,000×*g* for 30 min at 4 °C. Soluble fractions containing the His-tagged *cwh1* proteins were loaded on a Ni^2+^-NTA affinity column (TransGen, Beijing). After extensive washes with buffer A, the His-tagged proteins were eluted with buffer B (300 mM NaCl, 50 mM NaH_2_PO_4_, 250 mM imidazole, 10 mM Tris base, pH 8.0) and kept at − 20 °C.

Polypeptides were first separated by 12% polyacrylamide SDS-PAGE and then transferred onto a nitrocellulose membrane for Western blotting. His-tagged *cwh1* proteins were detected specifically with an antibody (THE His Tag Antibody, mAb, Mouse, Legend Biotech, Nanjing) used at a 1/1000 dilution. As a result, the two His-tagged *cwh1* proteins were successfully expressed through the *E. coli* system. Both proteins were primarily present in the soluble phase. Western blot analysis with anti-His antibodies revealed the presence of a single band at approximately 15 kDa (Fig. 3a), which was consistent with the protein size calculated based on amino acid sequence length.

The above two recombinant *cwh1* proteins were used to test the antibacterial activity against *E. coli* and *B. subtilis*, with methods referenced from Wu et al. [70]. Two strains of bacteria were cultured in LB medium overnight and subsequently resuspended in fresh LB medium and diluted to an OD_630 nm_ = 0.2, as measured by an absorbance microplate reader (BioTek, USA). The two recombinant *cwh1* proteins were diluted to 1 μM, 0.1 μM, and 0.01 μM. Then, 100 μL of each protein solution was mixed with 100 μL of the bacterial suspension in a 96-well microplate and incubated at 37 °C for 90 min. A mixture of 100 μL of buffer A (described above) and 100 μL of bacterial suspension was used as a negative control. Five replicates for each treatment and control were set. The increase in the OD_630 nm_ of each well within 90 min was measured.

### Transcriptome response of *cwh*-RNAi

Three *cwh*-RNAi strains of *C. montrouzieri* including CM-C1a-RNAi, CM-C1b-RNAi, and CM-C2-RNAi were constructed. Double-stranded RNA (dsRNA) primers were designed using the SnapDragon tool (http://www.flyrnai.org/cgi-bin/RNAi_find_primers.pl) and as shown in Additional file 1: Table S7. To avoid potential off-target effects, primers were further tested by alignment back to the *C. montrouzieri* genome using BLAST. The Promega T7 RiboMAX Express RNAi System was used to synthesize dsRNA according to the manufacturer’s protocol. The dsRNA purity and integrity were determined using agarose gel electrophoresis. As a control, green fluorescent protein (GFP)-dsRNA was also produced as described above. Injection of dsRNA (0.5 μL) into 4th instar larva was conducted using a micro-applicator. Insects injected with the same volume of GFP-dsRNA were set as controls. The efficiency of RNAi was confirmed after 24 h of injection using qPCR as described above.

Total RNA of qPCR-confirmed *cwh*-RNAi strains was used for transcriptome sequencing (Additional file 1: Table S3). RNA libraries were constructed and sequenced on an Illumina HiSeq X Ten platform with a 6-Gb sequencing depth. Three biological replicates were set for each *cwh*-RNAi strain as well as the GFP control. Clean reads were mapped to the *C. montrouzieri* genome using TopHat 2.1.1 [71]. Subsequently, Cufflinks v2.2.1 was used to obtain the mRNA sequences of each gene [71]. Differential expression analysis was performed with DESeq2 [72] according to the standard workflow.

### Detection of *cwh* genes in ladybird transcriptomes

A total of 38 ladybird species were selected for transcriptome sequencing (Additional file 1: Table S3), covering the 2/2 subfamilies and major tribes in Coccinellidae (according to the taxonomic system described by Escalona and Ślipiński) [73]. Adult individuals were collected in the field or taken from laboratory cultures and frozen in liquid nitrogen or at − 80 °C before RNA extraction. For most species, RNA was extracted from one individual. RNA libraries were constructed and sequenced on an Illumina HiSeq X Ten platform with a 6-Gb sequencing depth for each species. All reads were assembled with Trinity v2.8.4 [74] using default parameters. In consideration of frequent cross contamination, CroCo v1.1 [75] was used with default parameters to detect and remove the contaminating contigs in the transcriptome assemblies in each batch.

TransDecoder v5.5.0 (https://github.com/TransDecoder/) was used to identify the open reading frame per transcript. Redundancy at 90% identity of sequences within each species was eliminated using CD-HIT [76]. The “Hydrolase_2” domain-containing genes were identified as *cwh* genes. The glutamate catalytic domain of *cwh* protein sequences was predicted based on the studies of *Bacillus cereus* and *B. anthracis* [37, 38]. The tests for selection were performed on aligned and degapped nucleotide sequences of both ladybird *cwh1* and *cwh2*. Whole-gene nonsynonymous/synonymous ratio (dN/dS) calculations and statistical tests for positive or negative selection for individual codons were performed using SLAC in HyPhy [77].

### Detection of *cwh* homologs in eukaryotic genomes

To detect candidate eukaryotic *cwh* homologs, the *cwh* protein sequence of *C. montrouzieri* CM-C1a was used as a query to perform a BLASTp search against the NCBI NR database with a cut-off e-value of 10^− 10^. Significant hits were found in eukaryotic protein sequences from two species of nematodes, two water bears, two water fleas, and two springtails. To reduce the possibility of mistaking microbial contaminations for HGT, we examined primarily the genomes of *Daphnia magna*, *Folsomia candida*, *Orchesella cincta*, and *Hypsibius dujardini* since they were generated from long-read sequencing platforms and/or from sequencing of a single individual, and their complete coding sequences were predicted from RNA-Seq data. The presence of introns or flanking eukaryotic sequences was examined to provide further evidence for the presence of a eukaryotic *cwh* gene. Since the above putative *cwh* homologs were detected in both Nematoda and Panarthropoda, we performed a genome-wide investigation to study the distribution of *cwh* homologs in Edcysozoa. The CM-C1a protein sequence was used as a query to perform a tBLASTn search against all edcysozoan whole genome shotgun (WGS) assemblies deposited in NCBI with a cut-off e-value of 10^− 10^ and 90% coverage.

### Reconstruction of *cwh* gene trees

We performed a eukaryote-centered *cwh* phylogenetic analysis in order to clarify the relationship between eukaryotic *cwh* genes and their closely related non-eukaryotic genes. The *cwh* protein sequences from *C. montrouzieri*, *D. magna*, *F. candida*, *O. cincta*, and *H. dujardini* were used as a query to perform a BLASTp search against the NCBI NR database with cut-off e-value of 10^− 10^ and 90% coverage. About 10% representative hits from non-eukaryotes were selected and grouped with (1) the query sequences or (2) all putative eukaryotic *cwh* protein sequences extracted from WGS assemblies for further phylogenetic analysis. The aligned sequence matrices were generated by MAFFT v7.427 [78] with the L-INS-i algorithm and visually inspected. The highly variable C- and N-terminals in the matrix were trimmed before further phylogenetic analysis. IQ-TREE v1.6.12 [79] was used to select the best model of the alignments and to reconstruct the phylogenetic gene trees, with node support values estimated using the embedded ultrafast bootstrap approach (UFBoot). The phylogeny of all *cwh* genes from the genomes and transcriptomes of ladybird species was also reconstructed using MAFFT and IQ-TREE as described above.

### Phylogeny and divergence time estimate for ladybird species

Phylogenetic analysis was conducted on ladybirds and their Coleoptera outgroups. Nine species of Coleoptera outgroups from OrthoDB v10 and 13 from the Sequence Read Achieve (SRA) database were selected (Additional file 1: Table S3). The transcriptome data from SRA were assembled using Trinity as described above. Orthologous transcripts for each tested species were assigned using Orthograph v0.1 [80], with orthologous genes across 9 genomes in Coleoptera in the OrthoDB v10 database as reference. We used protein datasets of all 60 species in the subsequent analysis. To ensure that the genes to be used were single-copy genes in the whole Coleoptera group, we selected 885 genes with the following criteria: (i) these gene families should not include more than one ortholog in each species in the reference database; (ii) these genes were included in the Insecta OrthoDB v9 database and used as single-copy genes in BUSCO v3.0.2 [81], and the members of these genes were the same as those in the reference database; (iii) these genes could not be detected as duplicate genes by BUSCO in the published genome of *H. axyridis* [26]. Then, we used MAFFT v7.427 to align the gene sequences with the L-INS-i algorithm. Poorly aligned regions were removed using the “automated1” option of trimAl v1.4 [82]. The genes were further filtered to include those with more than 50 species with amino acids more than half the length of the alignments, and 819 aligned genes remained for further phylogenetic analysis.

To infer a partition scheme and proper substitution models, we subsequently used PartitionFinder v2.1.1 based on the corrected Akaike information criterion (AIC) with the rcluster algorithm. After the partition scheme was obtained, phylogenetic inference was conducted by RAxML v8.2.12 [83]. Branch support was calculated through a rapid bootstrap algorithm (-f a -x 12345 -p 12345 -# 1000 -m PROTGAMMA) with 1000 replicates.

Divergence time estimation was conducted by using MCMCTREE in PAML v4.8a [84]. The uncorrelated rate model (clock=2) was used, and 12 fossils were chosen to calibrate the clock (Additional file 1: Table S8) [85–96]. We constrained the maximum bound for the root (crown Coleoptera) to 323.2 MYA, according to the maximum age of the Coleoptera-Neuropterida split set by Zhang et al. [40], because no holometabolous insect was found in the Pennsylvanian, which began at this time.

Referring to the oldest beetle fossil *Coleopsis archaica* [97] and the time tree estimated by Zhang et al. [41], 295 MYA was set as the root age (crown Coleoptera) to calculate the prior on the overall substitution rate with the CODEML program in the PAML package. The ML estimates of the branch lengths were also calculated by CODEML. The LG+F+GAMMA model with four rate categories was used. The gamma-Dirichlet prior for overall rate for genes (rgene gamma) was set at G (1, 9.5), and the gamma-Dirichlet prior for sigma^2 (sigma2 gamma) was set at G (1, 4.5). Then, the MCMCTREE program in PAML estimated the divergence times by using a Monte Carlo algorithm. Burn-in was set as 8,000,000, meaning the first 8,000,000 iterations were discarded. Next, the samples were recorded every 8000 iterations until 10,000 samples were obtained. Another run was started from different seeds following the same steps. Finally, to check the convergence of the result, we used Tracer v1.7.1 [98] to ensure that all parameters were similar between two runs and that their effective sample sizes (ESSs) were all larger than 200.

## Supplementary Information

**Additional file 1: **Figure S1-S9, Tables S1-S7. Figure S1. Flanking genes of the eukaryotic *cwh* genes predicted from five high-quality genomes. Figure S2. Guanine-Cytosine contents of *cwh* coding sequences of the ladybird *Cryptolaemus montrouzieri*. Figure S3. Spatial expression of ladybird *cwh* genes. Figure S4. Temporal expression of ladybird *cwh* genes. Figure S5. Expression of ladybird *cwh* genes in response to bacterial infection. Figure S6. Phylogenetic tree of eukaryotic *cwh* genes identified from NCBI whole genome shotgun assemblies. Figure S7. Phylogenetic tree of Coccinellidae and their Coleoptera outgroups. Figure S8. Divergence time of Coccinellidae and their Coleoptera outgroups. Figure S9. Number of immunity-related genes in the published Coleoptera genomes. Table S1. Experimental design of *cwh* genes from ladybird genomes or transcriptomes. Table S2. Primers for ladybird *cwh* genes used for quantitative PCR. Table S3. Transcriptome data used in this study. Table S4. Information of the putative eukaryotic *cwh* genes detected from NCBI whole genome shotgun assemblies. Table S5. Estimation of selection pressures of *cwh1* and *cwh2* across Coccinellinae. Table S6. Primers for ladybird *cwh* used for cloning. Table S7. Primers for constructing *cwh*-RNAi strains of the ladybird *Cryptolaemus montrouzieri*.

**Additional file 2:** Raw Ct values of quantitative PCR.

**Additional file 3: **Multiple sequence alignment of the representative *cwh* protein sequences.

**Additional file 4: **Multiple sequence alignment of the ladybird *cwh* protein sequences.

## Data Availability

The genome of *C. montrouzieri* has been deposited in the NCBI BioProject PRJNA626074 [99]. The transcriptome of Coccinellidae species has been deposited in NCBI BioProject PRJNA626049 [100]. The transcriptome of *cwh*-RNAi strains of *C. montrouzieri* has been deposited in the NCBI BioProject PRJNA626073 [101]. The raw Ct data of qPCR can be found in Additional file 2. The alignment matrix of representative *cwh* protein sequences and all ladybird *cwh* protein sequences can be found in Additional files 3 and 4.

## References

[1] Soucy SM, Huang J, Gogarten JP (2015). Horizontal gene transfer: building the web of life. Nat Rev Genet.

[2] Husnik F, McCutcheon JP (2018). Functional horizontal gene transfer from bacteria to eukaryotes. Nat Rev Microbiol.

[3] McInerney JO (2017). Horizontal gene transfer is less frequent in eukaryotes than prokaryotes but can be important. Bioessays..

[4] Sieber KB, Bromley RE, Dunning Hotopp JC (2017). Lateral gene transfer between prokaryotes and eukaryotes. Exp Cell Res.

[5] Crisp A, Boschetti C, Perry M, Tunnacliffe A, Micklem G (2015). Expression of multiple horizontally acquired genes is a hallmark of both vertebrate and invertebrate genomes. Genome Biol.

[6] Danchin EG (2016). Lateral gene transfer in eukaryotes: tip of the iceberg or of the ice cube?. BMC Biol.

[7] Ku C, Martin WF (2016). A natural barrier to lateral gene transfer from prokaryotes to eukaryotes revealed from genomes: the 70% rule. BMC Biol.

[8] Koutsovoulos G, Kumar S, Laetsch DR, Stevens L, Daub J, Conlon C, Maroon H, Thomas F, Aboobaker AA, Blaxter M (2016). No evidence for extensive horizontal gene transfer in the genome of the tardigrade Hypsibius dujardini. P Natl Acad Sci USA..

[9] Schonknecht G, Weber AP, Lercher MJ (2014). Horizontal gene acquisitions by eukaryotes as drivers of adaptive evolution. Bioessays..

[10] Haegeman A, Jones JT, Danchin EGJ (2011). Horizontal gene transfer in nematodes: a catalyst for plant parasitism?. Mol Plant-Microbe Interact.

[11] Nakabachi A (2015). Horizontal gene transfers in insects. Curr Opin Insect Sci.

[12] Danchin EG, Guzeeva EA, Mantelin S, Berepiki A, Jones JT (2016). Horizontal gene transfer from bacteria has enabled the plant-parasitic nematode Globodera pallida to feed on host-derived sucrose. Mol Biol Evol.

[13] Wybouw N, Pauchet Y, Heckel DG, Van Leeuwen T (2016). Horizontal gene transfer contributes to the evolution of arthropod herbivory. Genome Biol Evol..

[14] Calderon-Cortes N, Quesada M, Watanabe H, Cano-Camacho H, Oyama K (2012). Endogenous plant cell wall digestion: a key mechanism in insect evolution. Annu Rev Ecol Evol Syst.

[15] Pauchet Y, Wilkinson P, Chauhan R, Ffrench-Constant RH (2010). Diversity of beetle genes encoding novel plant cell wall degrading enzymes. PLoS One.

[16] Pauchet Y, Heckel DG (2013). The genome of the mustard leaf beetle encodes two active xylanases originally acquired from bacteria through horizontal gene transfer. P Roy Soc Lond B Bio..

[17] Kirsch R, Gramzow L, Theissen G, Siegfried BD, Ffrench-Constant RH, Heckel DG, Pauchet Y (2014). Horizontal gene transfer and functional diversification of plant cell wall degrading polygalacturonases: key events in the evolution of herbivory in beetles. Insect Biochem Mol Biol.

[18] McKenna DD, Scully ED, Pauchet Y, Hoover K, Kirsch R, Geib SM, Mitchell RF, Waterhouse RM, Ahn SJ, Arsala D (2016). Genome of the Asian longhorned beetle (Anoplophora glabripennis), a globally significant invasive species, reveals key functional and evolutionary innovations at the beetle-plant interface. Genome Biol.

[19] McKenna DD, Shin S, Ahrens D, Balke M, Beza-Beza C, Clarke DJ, Donath A, Escalona HE, Friedrich F, Letsch H, et al. The evolution and genomic basis of beetle diversity. P Natl Acad Sci USA. 2019;116:24729–37. 10.1073/pnas.1909655116 PMC6900523 31740605

[20] Wybouw N, Dermauw W, Tirry L, Stevens C, Grbic M, Feyereisen R, Van Leeuwen T (2014). A gene horizontally transferred from bacteria protects arthropods from host plant cyanide poisoning. eLife..

[21] Moran NA, Jarvik T (2010). Lateral transfer of genes from fungi underlies carotenoid production in aphids. Science..

[22] Dunning Hotopp JC, Estes AM (2014). Biology wars: the eukaryotes strike back. Cell Host Microbe.

[23] Chou S, Bui NK, Russell AB, Lexa KW, Gardiner TE, LeRoux M, Vollmer W, Mougous JD (2012). Structure of a peptidoglycan amidase effector targeted to gram-negative bacteria by the type VI secretion system. Cell Rep.

[24] Ioannidis P, Lu Y, Kumar N, Creasy T, Daugherty S, Chibucos MC, Orvis J, Shetty A, Ott S, Flowers M (2014). Rapid transcriptome sequencing of an invasive pest, the brown marmorated stink bug Halyomorpha halys. BMC Genomics.

[25] Metcalf JA, Funkhouser-Jones LJ, Brileya K, Reysenbach AL, Bordenstein SR (2014). Antibacterial gene transfer across the tree of life. eLife.

[26] Baumann P. Diversity of prokaryote-insect associations within the Sternorrhyncha (psyllids, whiteflies, aphids, mealybugs). In. Bourtzis K, Miller T, editors. Insect Symbiosis, Volume 2. Florida: CRC Press; 2006. p. 23–46.

[27] Zhang L, Li S, Junyu Luo DP, Wu L, Li Y, Zhu X, Wang L, Zhang S, Cui J (2019). Chromosome-level genome assembly of the predator Propylea japonica to understand its tolerance to insecticides and high temperatures. Mol Ecol Res.

[28] Ando T, Matsuda T, Goto K, Hara K, Ito A, Hirata J, Yatomi J, Kajitani R, Okuno M, Yamaguchi K (2018). Repeated inversions within a pannier intron drive diversification of intraspecific colour patterns of ladybird beetles. Nat Commun.

[29] Gautier M, Yamaguchi J, Foucaud J, Loiseau A, Ausset A, Facon B, Gschloessl B, Lagnel J, Loire E, Parrinello H (2018). The genomic basis of color pattern polymorphism in the harlequin ladybird. Curr Biol.

[30] Moriyama R, Hattori A, Miyata S, Kudoh S, Makino S (1996). A gene (sleB) encoding a spore cortex-lytic enzyme from Bacillus subtilis and response of the enzyme to L-alanine-mediated germination. J Bacteriol.

[31] Hu K, Yang H, Liu G, Tan H (2007). Cloning and identification of a gene encoding spore cortex-lytic enzyme in Bacillus thuringiensis. Curr Microbiol.

[32] Faddeeva-Vakhrusheva A, Kraaijeveld K, Derks MFL, Anvar SY, Agamennone, V, Suring W, Kampfraath AA, Ellers J, Le Ngoc G, van Gestel CAM et al. Coping with living in the soil: the genome of the parthenogenetic springtail *Folsomia candida* BMC Genomics 2017, 18: 493. 10.1186/s12864-017-3852-x PMC5490193 28659179

[33] Faddeeva-Vakhrusheva A, Derks MFL, Anvar SY, Agamennone V, Suring W, Smit S, van Straalen CM, Roelofs D (2016). Gene family evolution reflects adaptation to soil environmental stressors in the genome of the collembolan Orchesella cincta. Genome Biol Evol.

[34] Lee BY, Choi BS, Kim MS, Park JC, Jeong CB, Han J, Lee JS (2019). The genome of the freshwater water flea Daphnia magna: a potential use for freshwater molecular ecotoxicology. Aquat Toxicol.

[35] Arakawa K, Arakawa K, Yoshida Y, Tomita M (2016). Genome sequencing of a single tardigrade Hypsibius dujardini individual. Sci Data.

[36] Ravenhall M, Škunca N, Lassalle F, Dessimoz C (2015). Inferring horizontal gene transfer. Plos Comput Biol.

[37] Jing X, Robinson HR, Heffron JD, Popham DL, Schubot FD (2012). The catalytic domain of the germination-specific lytic transglycosylase SleB from Bacillus anthracis displays a unique active site topology. Proteins..

[38] Li Y, Jin K, Setlow B, Setlow P, Hao B (2012). Crystal structure of the catalytic domain of the Bacillus cereus SleB protein, important in cortex peptidoglycan degradation during spore germination. J Bacteriol.

[39] Escalona HE, Zwick A, Li HS, Li J, Wang X, Pang H, Hartley D, Jermiin LS, Nedved O, Misof B (2017). Molecular phylogeny reveals food plasticity in the evolution of true ladybird beetles (Coleoptera: Coccinellidae: Coccinellini). BMC Evol Biol.

[40] Lidgard S, Crane PR (1990). Angiosperm diversification and Cretaceous floristic trends: a comparison of palynofloras and leaf macrofloras. Paleobiology..

[41] Zhang SQ, Che LH, Li Y, Dan L, Pang H, Slipinski A, Zhang P (2018). Evolutionary history of Coleoptera revealed by extensive sampling of genes and species. Nat Commun.

[42] McKenna DD, Sequeira AS, Marvaldi AE, Farrell BD (2009). Temporal lags and overlap in the diversification of weevils and flowering plants. P Natl Acad Sci USA..

[43] Wheat CW, Vogel H, Wittstock U, Braby MF, Underwood D, Mitchell-Olds T (2007). The genetic basis of a plant-insect coevolutionary key innovation. P Natl Acad Sci USA.

[44] Stroud JT, Losos JB (2016). Ecological opportunity and adaptive radiation. Annu Rev Ecol Evol Syst.

[45] McKenna DD, Wild AL, Kanda K, Bellamy CL, Beutel RG, Caterino MS, Farnum CW, Hawks DC, Ivie MA, Jameson ML (2015). The beetle tree of life reveals that Coleoptera survived end-Permian mass extinction to diversify during the Cretaceous terrestrial revolution. Syst Entomol.

[46] Robertson JA, Ślipiński A, Moulton M, Shockley FW, Giorgi A, Lord NP, McKenna DD, Tomaszewska W, Forrester J, Miller KB (2015). Phylogeny and classification of Cucujoidea and the recognition of a new superfamily Coccinelloidea (Coleoptera: Cucujiformia). Syst Entomol.

[47] Havill NP, Foottit RG, von Dohlen CD (2007). Evolution of host specialization in the Adelgidae (Insecta: Hemiptera) inferred from molecular phylogenetics. Mol Phylogenet Evol.

[48] Von Dohlen C (2000). Molecular data support a rapid radiation of aphids in the Cretaceous and multiple origins of host alternation. Biol J Linn Soc.

[49] Cunningham CB, Ji L, Wiberg RA, Shelton J, McKinney EC, Parker DJ, Meagher RB, Benowitz KM, Roy-Zokan EM, Ritchie MG (2015). The genome and methylome of a beetle with complex social behavior, Nicrophorus vespilloides (Coleoptera: Silphidae). Genome Biol Evol..

[50] Evans JD, McKenna D, Scully E, Cook SC, Dainat B, Egekwu N, Grubbs N, Lopez D, Lorenzen MD, Reyna SM (2018). Genome of the small hive beetle (Aethina tumida, Coleoptera: Nitidulidae), a worldwide parasite of social bee colonies, provides insights into detoxification and herbivory. Gigascience..

[51] Keeling CI, Yuen MM, Liao NY, Docking TR, Chan SK, Taylor GA, Palmquist DL, Jackman SD, Nguyen A, Li M (2013). Draft genome of the mountain pine beetle, Dendroctonus ponderosae Hopkins, a major forest pest. Genome Biol.

[52] Meyer JM, Markov GV, Baskaran P, Herrmann M, Sommer RJ, Rodelsperger C (2016). Draft genome of the scarab beetle Oryctes borbonicus on La Reunion Island. Genome Biol Evol..

[53] Richards S, Gibbs RA, Weinstock GM, Brown SJ, Denell R, Beeman RW, Gibbs R, Beeman RW, Brown SJ, Bucher G (2008). The genome of the model beetle and pest Tribolium castaneum. Nature..

[54] Schoville SD, Chen YH, Andersson MN, Benoit JB, Bhandari A, Bowsher JH, Brevik K, Cappelle K, Chen MM, Childers AK (2018). A model species for agricultural pest genomics: the genome of the Colorado potato beetle, Leptinotarsa decemlineata (Coleoptera: Chrysomelidae). Sci Rep.

[55] Thomas GWC, Dohmen E, Hughes DST, Murali SC, Poelchau M, Glastad K, Anstead CA, Ayoub NA, Batterham P, Bellair M (2020). Gene content evolution in the arthropods. Genome Biol.

[56] Zdybicka-Barabas A, Staczek S, Mak P, Skrzypiec K, Mendyk E, Cytrynska M (1828). Synergistic action of galleria mellonella apolipophorin III and lysozyme against gram-negative bacteria. Biochim Biophys Acta.

[57] Zdybicka-Barabas A, Mak P, Klys A, Skrzypiec K, Mendyk E, Fiolka MJ, Cytrynska M (1818). Synergistic action of galleria mellonella anionic peptide 2 and lysozyme against gram-negative bacteria. Biochim Biophys Acta.

[58] Hanson MA, Dostalova A, Ceroni C, Poidevin M, Kondo S, Lemaitre B (2019). Synergy and remarkable specificity of antimicrobial peptides in vivo using a systematic knockout approach. eLife..

[59] Vilcinskas A, Mukherjee K, Vogel H (2013). Expansion of the antimicrobial peptide repertoire in the invasive ladybird Harmonia axyridis. P Roy Soc Lond B Bio.

[60] Park J-W, Kim C-H, Rui J, Ryu K-H, Chai J-H, Hwang H-O, Kurokawa K, Ha N-C, Söderhäll I, Söderhäll K. Beetle immunity. In. Söderhäll K, editor.Invertebrate Immunity. New York: Springer; 2010. p. 163–180. 10.1007/978-1-4419-8059-5_9 21528698

[61] Vogel H, Schmidtberg H, Vilcinskas A (2017). Comparative transcriptomics in three ladybird species supports a role for immunity in invasion biology. Dev Comp Immunol.

[62] Wittekind M, Schuch R (2016). Cell wall hydrolases and antibiotics: exploiting synergy to create efficacious new antimicrobial treatments. Curr Opin Microbiol.

[63] Yoshida Y, Koutsovoulos G, Laetsch DR, Stevens L, Kumar S, Horikawa DD, Ishino K, Komine S, Kunieda T, Tomita M, Yoshida Y (2017). Comparative genomics of the tardigrades Hypsibius dujardini and Ramazzottius varieornatus. Plos Biol.

[64] Jones P, Binns D, Chang HY, Fraser M, Li WZ, McAnulla C, McWilliam H, Maslen J, Mitchell A, Nuka G (2014). InterProScan 5: genome-scale protein function classification. Bioinformatics..

[65] Armenteros JJA, Tsirigos KD, Sonderby CK, Petersen TN, Winther O, Brunak S, von Heijne G, Nielsen H (2019). SignalP 5.0 improves signal peptide predictions using deep neural networks. Nat Biotechnol.

[66] Lu J, Chen S, Guo M, Ye C, Qiu B, Yang C, Pan H (2018). Selection of appropriate reference genes for RT-qPCR analysis in Propylea japonica (Coleoptera: Coccinellidae). Plos One.

[67] Qu C, Wang R, Che W, Zhu X, Li F, Luo C (2018). Selection and evaluation of reference genes for expression analysis using quantitative real-time PCR in the Asian ladybird Harmonia axyridis (Coleoptera: Coccinellidae). Plos One.

[68] Pan C, Zhang Y, Pang H (2016). Selection of the reference genes for gene expression studies in Cryptolaemus montrouzieri Mulsant by qRT-PCR. J Environ Entomol.

[69] Livak KJ, Schmittgen TD (2001). Analysis of relative gene expression data using real-time quantitative PCR and the 2(T)(−Delta Delta C) method. Methods..

[70] Wu B, Liu Z, Zhou L, Ji G, Yang A (2015). Molecular cloning, expression, purification and characterization of vitellogenin in scallop Patinopecten yessoensis with special emphasis on its antibacterial activity. Dev Comp Immunol.

[71] Trapnell C, Roberts A, Goff L, Pertea G, Kim D, Kelley DR, Pimentel H, Salzberg SL, Rinn JL, Pachter L (2012). Differential gene and transcript expression analysis of RNA-seq experiments with TopHat and Cufflinks. Nat Protoc.

[72] Love MI, Huber W, Anders S (2014). Moderated estimation of fold change and dispersion for RNA-seq data with DESeq2. Genome Biol.

[73] Escalona HE, Ślipiński A (2012). Generic revision and phylogeny of Microweiseinae (Coleoptera: Coccinellidae). Syst Entomol.

[74] Grabherr MG, Haas BJ, Yassour M, Levin JZ, Thompson DA, Amit I, Adiconis X, Fan L, Raychowdhury R, Zeng QD (2011). Full-length transcriptome assembly from RNA-Seq data without a reference genome. Nat Biotechnol.

[75] Simion P, Belkhir K, Francois C, Veyssier J, Rink JC, Manuel M, Philippe H, Telford MJ (2018). A software tool ‘CroCo’ detects pervasive cross-species contamination in next generation sequencing data. BMC Biol.

[76] Li WZ, Godzik A (2006). CD-HIT: a fast program for clustering and comparing large sets of protein or nucleotide sequences. Bioinformatics..

[77] Pond SLK, Frost SDW, Muse SV (2005). HyPhy: hypothesis testing using phylogenies. Bioinformatics..

[78] Katoh K, Standley DM (2013). MAFFT multiple sequence alignment software version 7: improvements in performance and usability. Mol Biol Evol.

[79] Nguyen LT, Schmidt HA, Haeseler AV, Minh BQ (2015). IQ-TREE: a fast and effective stochastic algorithm for estimating maximum-likelihood phylogenies. Mol Biol Evol.

[80] Petersen M, Meusemann K, Donath A, Dowling D, Liu SL, Peters RS, Podsiadlowski L, Vasilikopoulos A, Zhou X, Misof B (2017). Orthograph: a versatile tool for mapping coding nucleotide sequences to clusters of orthologous genes. BMC Bioinformatics.

[81] Waterhouse RM, Seppey M, Simao FA, Manni M, Ioannidis P, Klioutchnikov G, Kriventseva EV, Zdobnov EM (2018). BUSCO applications from quality assessments to gene prediction and phylogenomics. Mol Biol Evol.

[82] Capella-Gutierrez S, Silla-Martinez JM, Gabaldon T trimAl: a tool for automated alignment trimming in large-scale phylogenetic analyses Bioinformatics 2009, 25:1972–1973. 10.1093/bioinformatics/btp348 PMC2712344 19505945

[83] Stamatakis A (2014). RAxML version 8: a tool for phylogenetic analysis and post-analysis of large phylogenies. Bioinformatics..

[84] Yang ZH (2007). PAML 4: phylogenetic analysis by maximum likelihood. Mol Biol Evol.

[85] Wickham HF (1913). The Princeton collection of fossil beetles from Florissant. Ann Entomol Soc Am.

[86] Szawaryn K (2019). Unexpected diversity of whitefly predators in Eocene Baltic amber - new fossil Serangium species (Coleoptera: Coccinellidae). Zootaxa..

[87] Kirejtshuk AG, Nel A (2012). The oldest representatives of the family Coccinellidae (Coleoptera: Polyphaga) from the lowermost Eocene Oise amber (France). Zoosystematica Rossica.

[88] Kirejtshuk AG, Azar D (2009). Taxonomic names, in new beetles of Polyphaga (Coleoptera, Polyphaga) from lower Cretaceous Lebanese amber. Denisia..

[89] Wang B, Ma JY, McKenna DD, Yan EV, Zhang HC, Jarzembowski EA (2014). The earliest known longhorn beetle (Cerambycidae: Prioninae) and implications for the early evolution of Chrysomeloidea. J Syst Palaeontol.

[90] Arnoldi LV. Rhynchopora. in Mezozoiskie zhestkokryiye. Akademiya Nauk SSSR, Trudy Paleontologicheskogo Instituta 1977, 161:142–176.

[91] Martynov AV. K Poznaniyu Iskopaemykh Nasekomykh Yurskikh Slantsev Turkestana. 5. O Nekotorykh Formakh Zhukov (Coleoptera) [to the knowledge of fossil insects from Jurassic beds in Turkestan 5. On some interesting Coleoptera]. Ezhegodnik Russkogo Paleontologicheskogo Obshestva 1926, 5:1–39.

[92] Medvedev LN (1969). New Mesozoic Coleoptera (Cucujoidea) of Asia. Paleontol J.

[93] Deng CS, Ślipiński A, Ren D, Pang H (2017). The oldest dermestid beetle from the middle Jurassic of China (Coleoptera: Dermestidae). Anna Zool.

[94] Nikolajev GV, Wang B, Liu Y, Zhang HC (2011). Stag beetles from the Mesozoic of Inner Mongolia, China (Scarabaeoidea: Lucanidae). Acta Palaeontol Sin.

[95] Alekseev AV (1993). Jurassic and lower Cretaceous Buprestidae (Coleoptera) from Eurasia. Paleontol J.

[96] Ponomarenko AG. Suborder Adephaga, Polyphaga Incertae Sedis, Infraorder Staphyliniformia, in Mezozoiskie zhestkokryiye (Mesozoic Coleoptera). Akademiya Nauk SSSR, Trudy Paleontologicheskogo Instituta 1977, 161:17–119.

[97] Kirejtshuk AG, Poschmann M, Prokop J, Garrouste R, Nel A (2014). Evolution of the elytral venation and structural adaptations in the oldest Palaeozoic beetles (Insecta: Coleoptera: Tshekardocoleidae). J Syst Palaeontol.

[98] Drummond AJ, Ho SY, Phillips MJ, Rambaut A (2006). Relaxed phylogenetics and dating with confidence. PLoS Biol.

[99] Li HS. *Cryptolaemus montrouzieri* isolate: SYSU2018 Genome sequencing and assembly. NCBI WGS. PRJNA626074. https://www.ncbi.nlm.nih.gov/bioproject/PRJNA626074. Accessed 2 July 2020.

[100] Li HS. Transcriptome of different Coccinellidae species. NCBI SRA. PRJNA626049. https://www.ncbi.nlm.nih.gov/bioproject/PRJNA626049. Accessed 17 Apr 2020.

[101] Li HS. Transcriptome of cwh-RNAi strains of *Cryptolaemus montrouzieri*. NCBI SRA. PRJNA626073. https://www.ncbi.nlm.nih.gov/bioproject/PRJNA626073. Accessed 18 Apr 2020.

