# Supplementary material for: Horizontally acquired antibacterial genes associated with adaptive radiation of ladybird beetles
Source: BMC Biol. 2021 Jan 14;19:7. doi: 10.1186/s12915-020-00945-7 (PMC7807722; doi:10.1186/s12915-020-00945-7)
Supplement: Supplementary file 1 — Additional file 1: Figure S1-S9, Tables S1-S7. Figure S1. Flanking genes of the eukaryotic cwh genes predicted from five high-quality genomes. Figure S2. Guanine-Cytosine contents of cwh coding sequences of the ladybird Cryptolaemus montrouzieri. Figure S3. Spatial expression of ladybird cwh genes. Figure S4. Temporal expression of ladybird cwh genes. Figure S5. Expression of ladybird cwh genes in response to bacterial infection. Figure S6. Phylogenetic tree of eukaryotic cwh genes identified from NCBI whole genome shotgun assemblies. Figure S7. Phylogenetic tree of Coccinellidae and their Coleoptera outgroups. Figure S8. Divergence time of Coccinellidae and their Coleoptera outgroups. Figure S9. Number of immunity-related genes in the published Coleoptera genomes. Table S1. Experimental design of cwh genes from ladybird genomes or transcriptomes. Table S2. Primers for ladybird cwh genes used for quantitative PCR. Table S3. Transcriptome data used in this study. Table S4. Information of the putative eukaryotic cwh genes detected from NCBI whole genome shotgun assemblies. Table S5. Estimation of selection pressures of cwh1 and cwh2 across Coccinellinae. Table S6. Primers for ladybird cwh used for cloning. Table S7. Primers for constructing cwh-RNAi strains of the ladybird Cryptolaemus montrouzieri. [file 12915_2020_945_MOESM1_ESM.docx]

Additional file 1 for

Potential role of horizontally acquired antibacterial genes in the adaptive radiation of ladybird beetles

Hao-Sen Li^1#^, Xue-Fei Tang^1#^, Yu-Hao Huang^1#^, Ze-Yu Xu^1^, Mei-Lan Chen^1,2^, Xue-Yong Du^1^, Bo-Yuan Qiu^1^, Pei-Tao Chen^1^, Wei Zhang^1^, Adam Ślipiński^3^, Hermes E. Escalona^3^, Robert M. Waterhouse^4^, Andreas Zwick^3^, Hong Pang^1^*

1 State Key Laboratory of Biocontrol, School of Life Sciences / School of Ecology, Sun Yat-sen University, Guangzhou 510275, China

2 School of Environment and Life Science, Nanning Normal University, Nanning 530001, China

3 Australian National Insect Collection, CSIRO, GPO Box 1700, Canberra ACT 2601, Australia

4 Department of Ecology and Evolution, University of Lausanne and Swiss Institute of Bioinformatics, 1015 Lausanne, Switzerland

# theses authors contributed equally to this work

* corresponding author: Hong Pang

**Email:** lsshpang@mail.sysu.edu.cn


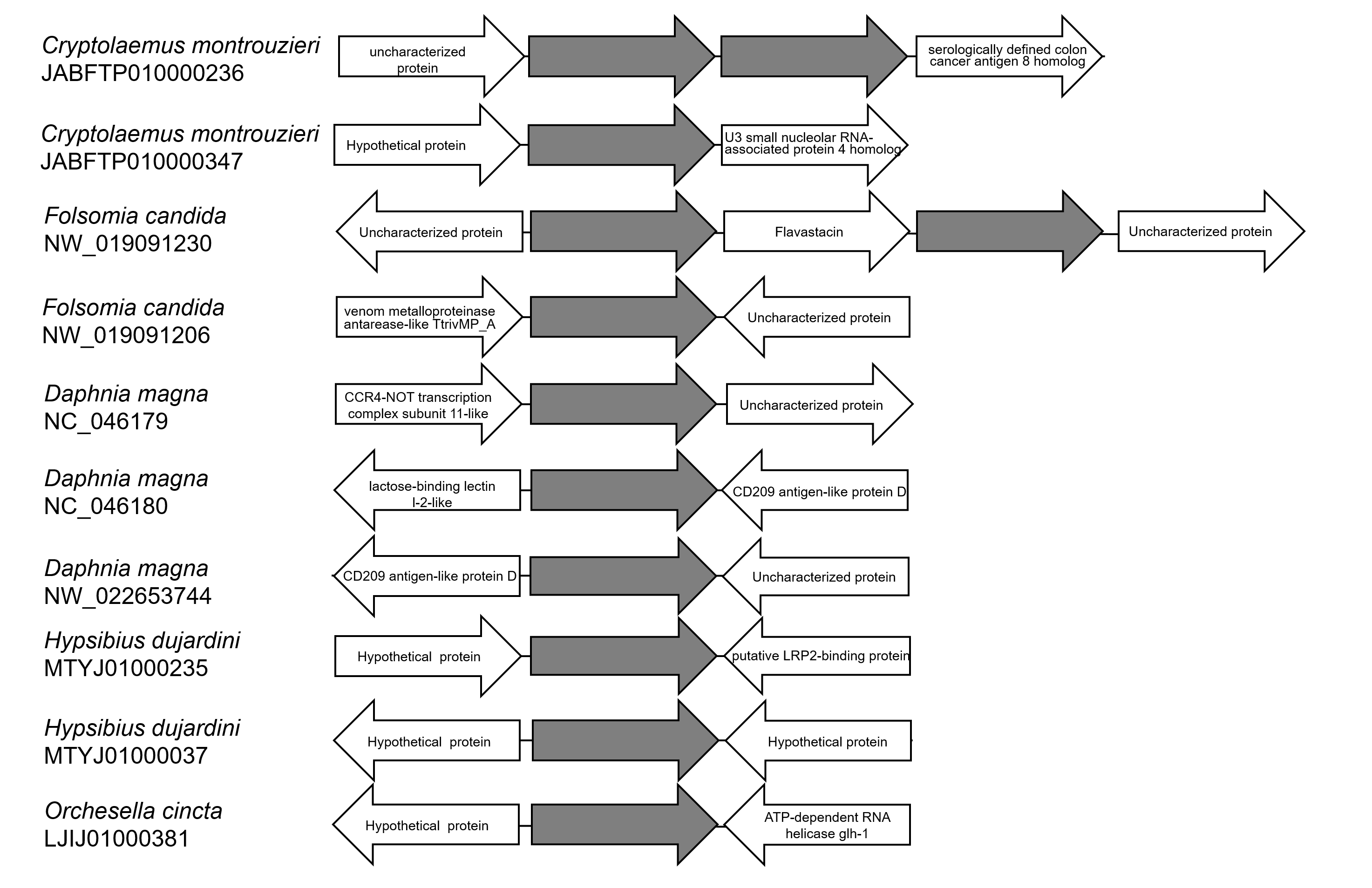


**Figure S1** Flanking genes of the eukaryotic *cwh* genes predicted from five high-quality genomes. The contigs/scaffolds containing putative eukaryotic *cwh* genes are shown. The *cwh* genes are coloured in grey. BLAST searches suggests that these flanking genes are closely related to eukaryotic genes.


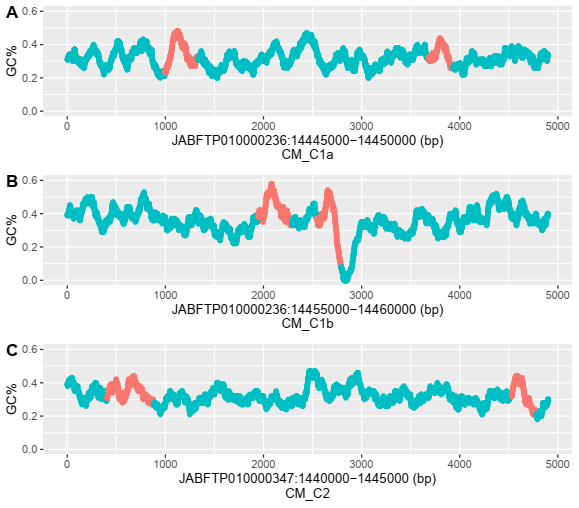


**Figure S2** Guanine-Cytosine contents (GC%) (100 bp sliding window) of *cwh* coding sequences (red) of the ladybird *Cryptolaemus montrouzieri* (A: CM-C1a; B: CM-C1b; C: CM-C2) are compared to their introns and flanking sequences (blue).


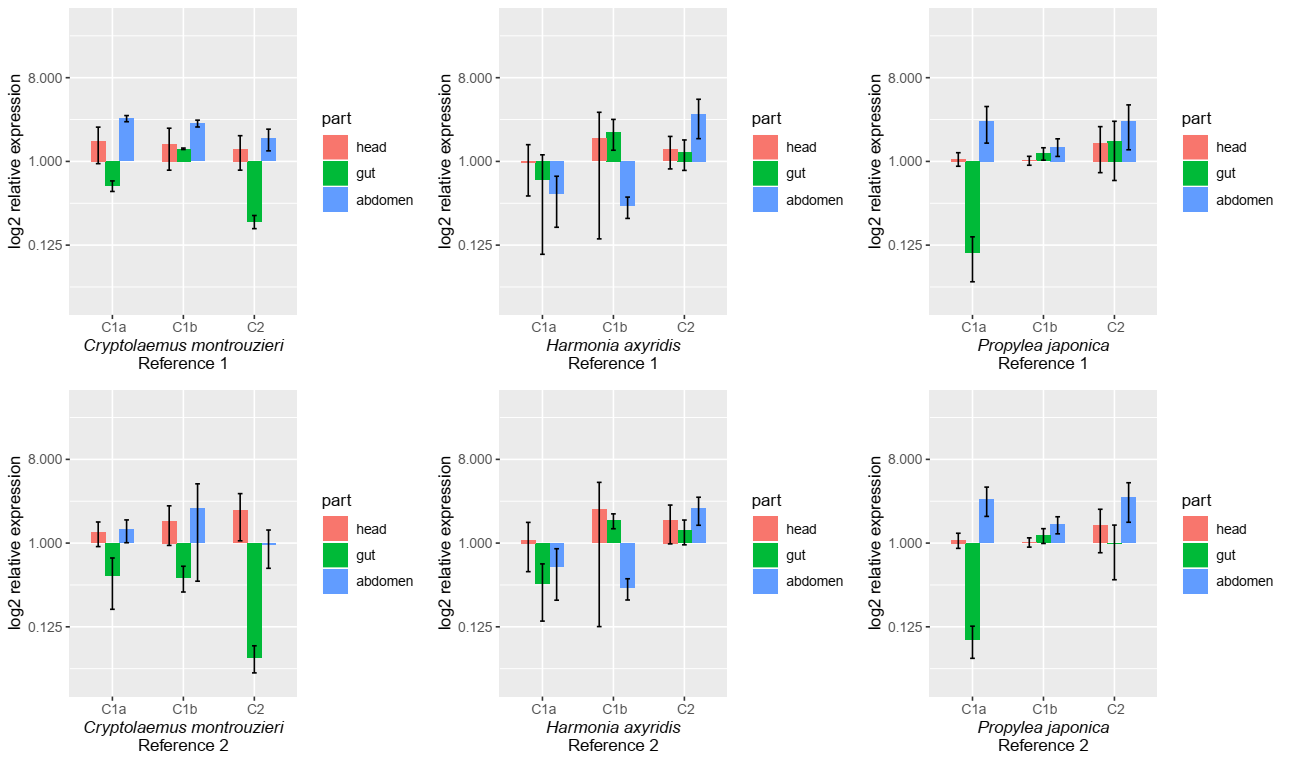


**Figure S3** Spatial expression of ladybird *cwh* genes. The expression patterns of *cwh* genes (C1a, C1b and C2, or C1 and C2) in different body parts were tested in *Cryptolaemus montrouzieri, Harmonia axyridis* and *Propylea japonica*. Expression levels of *cwh* were normalized to those of two reference genes (see details of reference genes in Table S2). Relative expression of each *cwh* gene in comparison to the average level of that gene in head was analyzed by the 2^-ΔΔCt^ method to calculate the fold changes. Error bars show ± standard errors with five biological replicates. No significant difference between treatments (*p* <0.05) was detected.


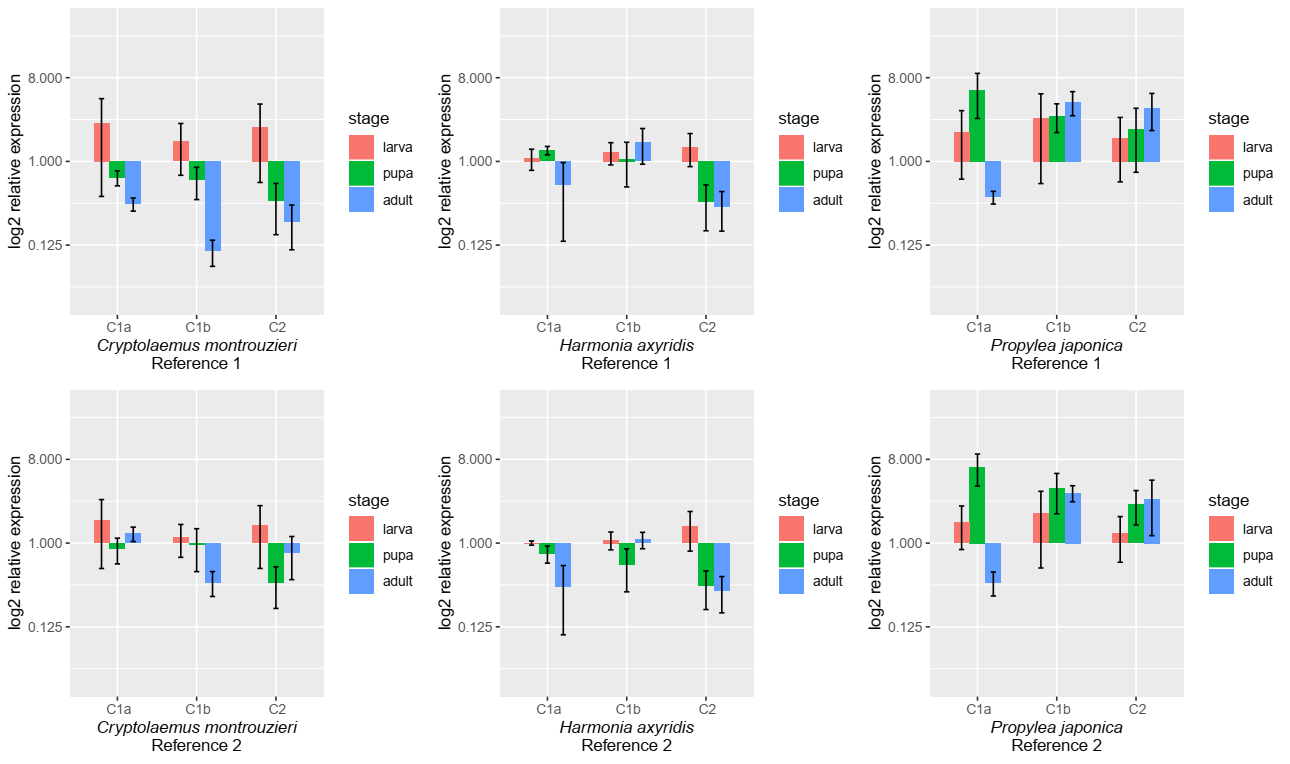


**Figure S4** Temporal expression of ladybird *cwh* genes. The expression patterns of *cwh* genes in different life stages were tested in *Cryptolaemus montrouzieri, Harmonia axyridis* and *Propylea japonica*. Expression levels of *cwh* were normalized to those of two reference genes (see details of reference genes in Supplementary Table 2). Relative expression of each *cwh* gene in comparison to the average level of that gene in larva was analyzed by the 2^-ΔΔCt^ method to calculate the fold changes. Error bars show ± standard errors with five biological replicates. No significant difference between treatments (*p* <0.05) was detected.


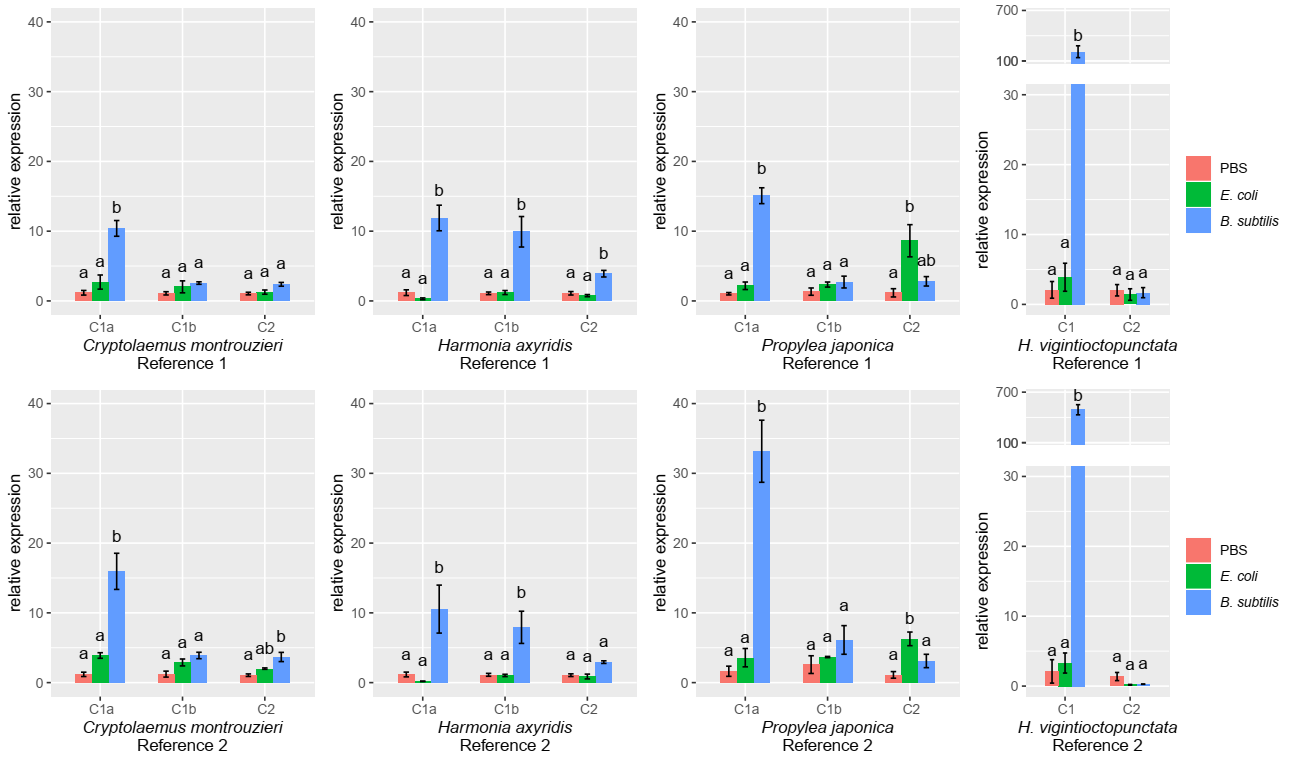


**Figure S5** Evidence of the antibacterial activity of ladybird *cwh* genes: upregulation of *cwh* in response to bacterial infection. The expression patterns of *cwh* genes (C1a, C1b and C2, or C1 and C2) in response to bacterial infection (*E. coli* and *B. subtilis*) were tested in *Cryptolaemus montrouzieri, Harmonia axyridis*, *Propylea japonica* and *Henosepilachna vigintioctopunctata*. PBS treatments were used as control. Expression levels of *cwh* were normalized to those of two reference genes (see details of two reference genes in Supplementary Table 2). Relative expression of each *cwh* gene in comparison to the average level of that gene in PBS treatment was analyzed by the 2^-ΔΔCt^ method to calculate the fold changes. Error bars show ± standard errors with five biological replicates. Bars with the same letter are not significantly different (*p* ≥0.05).


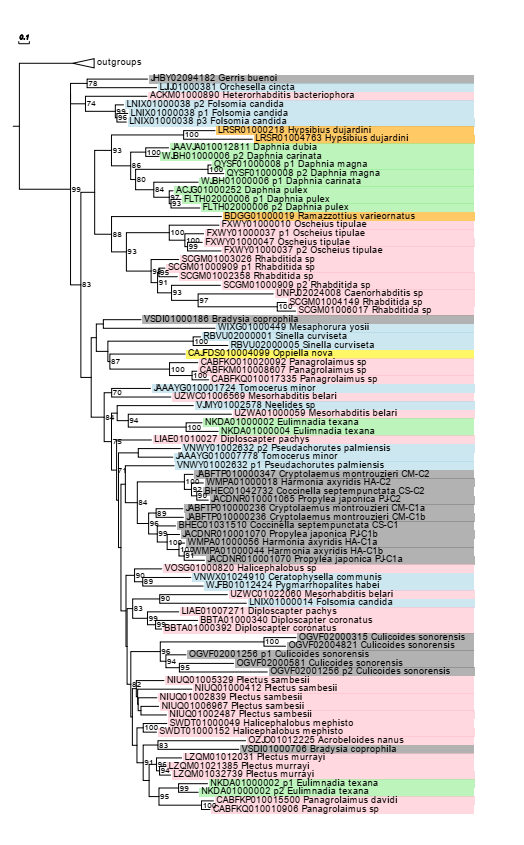


**Figure S6** The phylogenetic tree of eukaryotic *cwh* genes identified from NCBI whole genome shotgun (WGS) assemblies. Redundancy at 90% identity at the protein level between sequences was eliminated using CD-HIT. Sequences containing stop codon were also removed from analysis. The tree was reconstructed by the maximum likelihood method and rooted by the representative bacterial *cwh* genes. Only node supports >70 are shown. Contig/scaffold and species names of genes are colour-coded according to taxonomic group of organisms (Pink: Nematoda; orange: Tardigrada; yellow: Chelicerata; blue: Branchiopoda; green: Collembola; grey: Insecta).


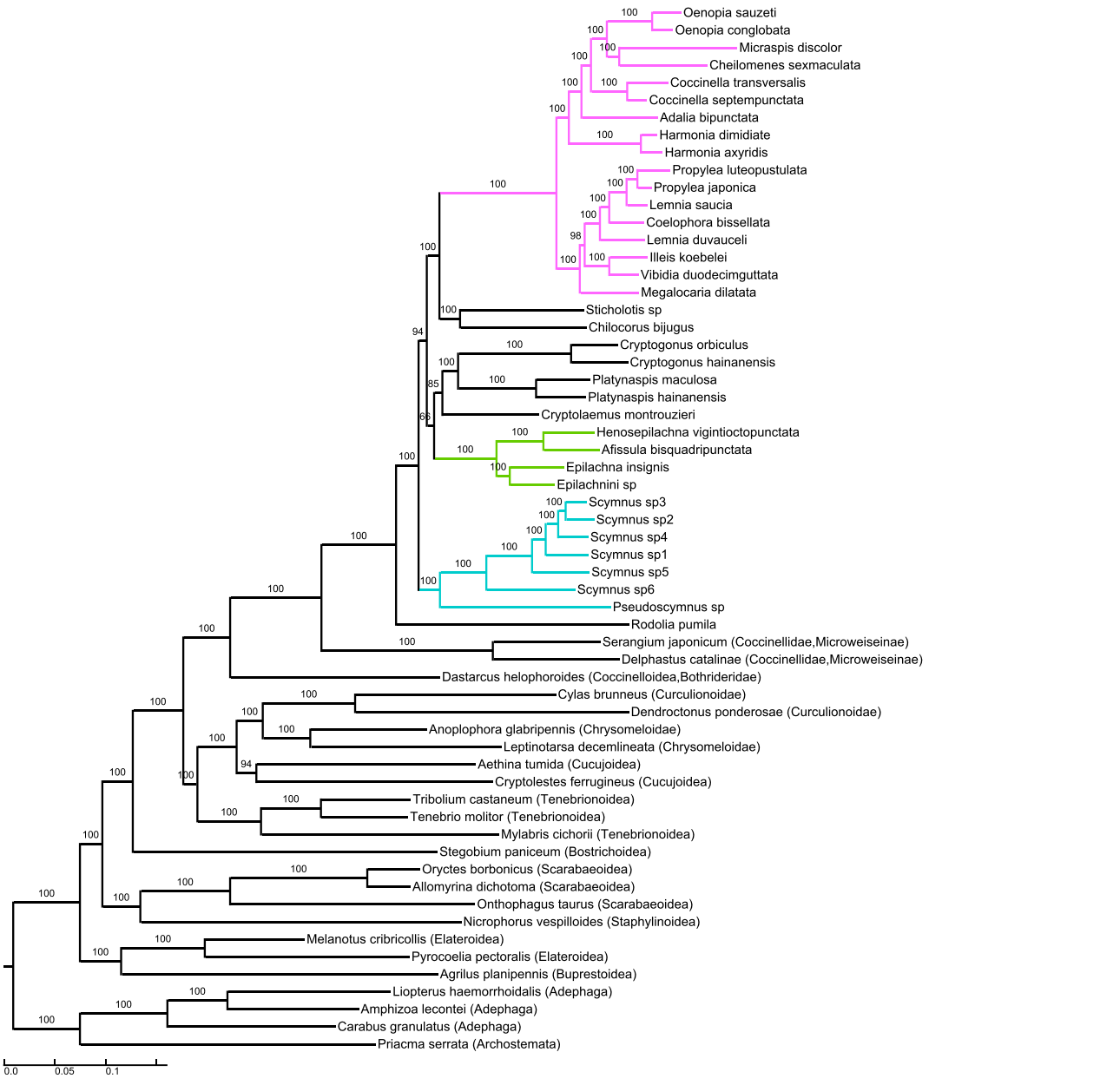


**Figure S7** Phylogenetic tree of Coccinellidae and their Coleoptera outgroups reconstructed by 819 single-copy genes and maximum likelihood method. Node supports represent bootstrap values. Three major monophyletic clades of Coccinellinae include Coccinellini (red), Epilachini (green) and Scymnini (blue) are colour-coded.


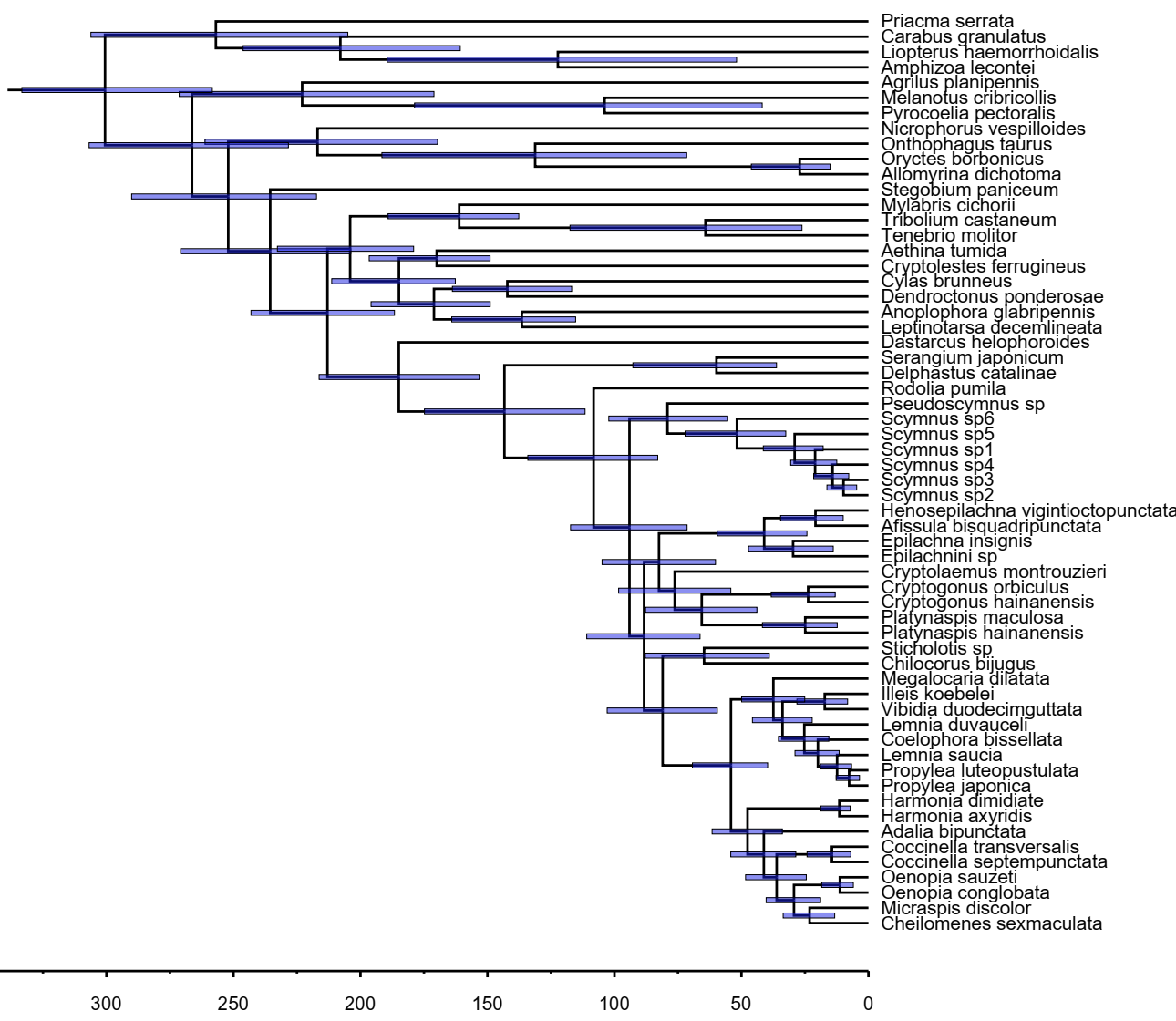


**Figure S8** Phylogenetic tree of Coccinellidae and their Coleoptera outgroups reconstructed by 819 single-copy genes and maximum likelihood method. Divergence time was estimated using MCMCTREE method and 12 fossils for calibration. Horizontal bars represent 95% confidence intervals.


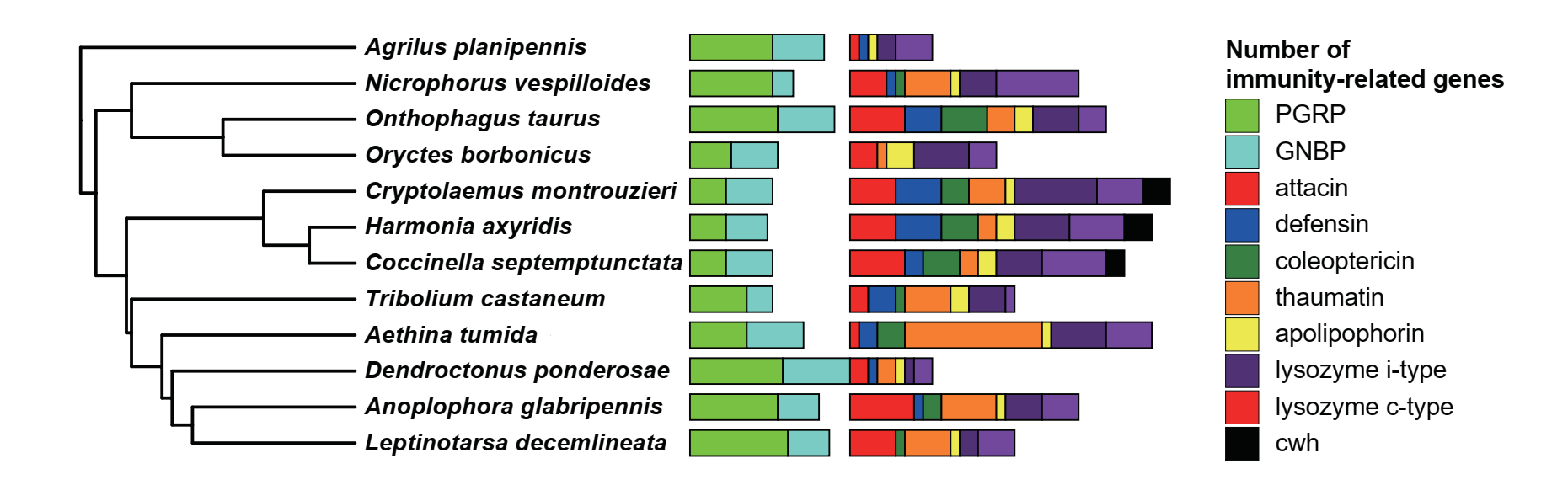


**Figure S9** Number of immunity-related genes involving in bacteria recognition (left) and defense execution (right) in the published Coleoptera genomes [18, 19, 28, 48-54]. Species phylogenetic tree was adapted from Mckenna et al. 2019 [19].

**Table S1** The *cwh* genes from ladybird genomes (G) or transcriptomes (T) for analyses of genome characteristics (GENO), expression in different body parts (PART) and developmental stages (STAGE), expression during bacterial infection by injection (INJECT), recombinant expression (EXPRE), antibacterial activity (ANTIB) and RNAi strain construction (RNAI).

| Species | G/T | Gene | Gene ID | GENO | PART | STAGE | INJECT | EXPRE | ANTIB | RNAI |
| --- | --- | --- | --- | --- | --- | --- | --- | --- | --- | --- |
| *Cryptolaemus*  *montrouzieri* | G, T | *cwh1* | CM-C1a | Done | Done | Done | Done | Done | Done | Done |
|  |  | *cwh1* | CM-C1b | Done | Done | Done | Done | - | - | Done |
|  |  | *cwh2* | CM-C2 | Done | Done | Done | Done | - | - | Done |
| *Harmonia*  *axyridis* | G, T | *cwh1* | HA-C1a | Done | Done | Done | Done | - | - | - |
|  |  | *cwh1* | HA-C1b | Done | Done | Done | Done | Done | Done | - |
|  |  | *cwh2* | HA-C2 | Done | Done | Done | Done | - | - | - |
| *Coccinella*  *septempunctata* | G, T | *cwh1* | CS-C1 | Done | - | - | - | - | - | - |
|  |  | *cwh2* | CS-C2 | Done | - | - | - | - | - | - |
| *Propylea*  *japonica* | G, T | *cwh1* | PJ-C1a | Done | Done | Done | Done | - | - | - |
|  |  | *cwh1* | PJ-C1b | Done | Done | Done | Done | - | - | - |
|  |  | *cwh2* | PJ-C2 | Done | Done | Done | Done | - | - | - |
| *Henosepilachna*  *vigintioctopunctata* | T | *cwh1* | HV-C1 | - | - | - | Done | - | - | - |
|  |  | *cwh2* | HV-C2 | - | - | - | Done | - | - | - |

**Table S2** Primers for ladybird *cwh* genes used for quantitative PCR (qPCR). Sequences are given for the forward (F) and reverse (R) primers and the annealing temperature used for PCR (*T*m) with each primer pair.

| Species | Gene ID | F/R | Primer sequence (5’ - 3’) | *T*m |
| --- | --- | --- | --- | --- |
| *Cryptolaemus*  *montrouzieri* | CM-C1a | F | GGCAGTAAAATCGGGAATGT | 55 |
|  |  | R | CGGAAGAAAGAGGAGCATC |  |
|  | CM-C1b | F | TTTATGATGCTCCTCTTTCTGAT |  |
|  |  | R | AACTGGTGATTTCCGATTTTC |  |
|  | CM-C2 | F | CAGTGAAAATGGACGATAAGG |  |
|  |  | R | TAATAACCCAAGCCACCCA |  |
|  | Reference 1:  CM-RPS23 | F | ACAAGGGCTGCTGGACTGAT |  |
|  |  | R | CCTTCGGAGGAAAGGCTTCA |  |
|  | Reference 2:  CM-β-tubulin | F | CACGGAAGGTACTTGACTGTTG |  |
|  |  | R | GCTGCTGTTCTTGTTTTGGATG |  |
| *Harmonia*  *axyridis* | HA-C1a | F | GTCTGTCGCCAACCTTACC | 60 |
|  |  | R | TGGTCACAACCACCAGTAGG |  |
|  | HA-C1b | F | AACCGTCAACATTGGGGAGG |  |
|  |  | R | CATTGCGACCATTCCAGCAC |  |
|  | HA-C2 | F | TGGGGAGGTAGCGTAATTGG |  |
|  |  | R | GCTTGCTGGGGCATCATAGA |  |
|  | Reference 1:  HA-RPS13 | F | ACAGACGAAGTGTCCCAACA |  |
|  |  | R | CCTGAGCAACTCCAAGGGAAT |  |
|  | Reference 2:  HA-RPL28 | F | CAGAACCTAGCAACCTCACCA |  |
|  |  | R | AGGTCTCTGACAGACTACGGT |  |
| *Propylea*  *japonica* | PJ-C1a | F | AGCCTTGACTGATAGAGAAA | 55 |
|  |  | R | GCTTCCACCCCAATAAGAC |  |
|  | PJ -C1b | F | ATGCTGGAATGATGGTGATA |  |
|  |  | R | TTTAGGATTGTTGTAATGATCGC |  |
|  | PJ-_C2 | F | TATTGGGTCTGTCTGTCGTA |  |
|  |  | R | GCATTGGGGCATCATAAAT |  |
|  | Reference 1:  PJ-RPS18 | F | CGCTGGTGATTCCAGATAAA |  |
|  |  | R | GACGACCTACACCTTTGATG |  |
|  | Reference 2:  PJ-EF1α | F | CTGGAAAGACCACAGAAGAAA |  |
|  |  | R | GAGGAGGGAATTCTTGGAAAG |  |
| *Henosepilachna*  *vigintioctopunctata* | HV-C1 | F | GAATGGGTGGCTTGGGTGAT | 60 |
|  |  | R | CTAGGCTCAGTGACGTCGAA |  |
|  | HV-C2 | F | GAACCTGTTGAGGGGCAGAT |  |
|  |  | R | CACCAATTTTGCTTCCACCC |  |
|  | Reference 1:  HV-GADPH | F | ACTGCCGATGTTTCTGTTGT |  |
|  |  | R | GCTTCTTTGACTGCCTTTTTGA |  |
|  | Reference 2:  HV-actin | F | CTCCGTGTAGCCCCAGAAG |  |
|  |  | R | GGTCATCTTTTCCCTGTTAGCC |  |

**Table S3** Transcriptome data used for 1. study of transcriptome response of *cwh*-RNAi of *Cryptolaemus montrouzieri* and 2. detection of ladybird *cwh* genes and species phylogeny reconstruction.

| Transcriptome response of *cwh*-RNAi | | | |  |
| --- | --- | --- | --- | --- |
| cwh RNAi-strain | Replicate | Species | SRA accession |  |
| CM-C1a-RNAi | R1 | *Cryptolaemus montrouzieri* | SRR11566201 |  |
| CM-C1a-RNAi | R2 | *Cryptolaemus montrouzieri* | SRR11566200 |  |
| CM-C1a-RNAi | R3 | *Cryptolaemus montrouzieri* | SRR11566199 |  |
| CM-C1b-RNAi | R1 | *Cryptolaemus montrouzieri* | SRR11566206 |  |
| CM-C1b-RNAi | R2 | *Cryptolaemus montrouzieri* | SRR11566205 |  |
| CM-C1b-RNAi | R3 | *Cryptolaemus montrouzieri* | SRR11566202 |  |
| CM-C2-RNAi | R1 | *Cryptolaemus montrouzieri* | SRR11566198 |  |
| CM-C2-RNAi | R2 | *Cryptolaemus montrouzieri* | SRR11566197 |  |
| CM-C2-RNAi | R3 | *Cryptolaemus montrouzieri* | SRR11566196 |  |
| GFP control | R1 | *Cryptolaemus montrouzieri* | SRR11566195 |  |
| GFP control | R2 | *Cryptolaemus montrouzieri* | SRR11566204 |  |
| GFP control | R3 | *Cryptolaemus montrouzieri* | SRR11566203 |  |
| Ladybird *cwh* detection and species phylogeny | | | |  |
| Subfamily | Tribe | Species | SRA accession |  |
| Coccinellinae | Coccinellini | *Oenopia sauzeti* | SRR11576248 |  |
| Coccinellinae | Coccinellini | *Oenopia conglobata* | SRR11576247 |  |
| Coccinellinae | Coccinellini | *Micraspis discolor* | SRR11576236 |  |
| Coccinellinae | Coccinellini | *Cheilomenes sexmaculata* | SRR11576225 |  |
| Coccinellinae | Coccinellini | *Coccinella transversalis* | SRR11576220 |  |
| Coccinellinae | Coccinellini | *Coccinella septempunctata* | SRR11576219 |  |
| Coccinellinae | Coccinellini | *Adalia bipunctata* | SRR11576248 |  |
| Coccinellinae | Coccinellini | *Harmonia dimidiata* | SRR11576218 |  |
| Coccinellinae | Coccinellini | *Harmonia axyridis* | SRR9649801 |  |
| Coccinellinae | Coccinellini | *Propylea luteopustulata* | SRR11576217 |  |
| Coccinellinae | Coccinellini | *Propylea japonica* | SRR9649789 |  |
| Coccinellinae | Coccinellini | *Lemnia saucia* | SRR11576216 |  |
| Coccinellinae | Coccinellini | *Coelophora bissellata* | SRR11576215 |  |
| Coccinellinae | Coccinellini | *Lemnia duvauceli* | SRR11576246 |  |
| Coccinellinae | Psylloborini | *Illeis koebelei* | SRR11576245 |  |
| Coccinellinae | Psylloborini | *Vibidia duodecimguttata* | SRR11576244 |  |
| Coccinellinae | Coccinellini | *Megalocaria dilatata* | SRR11576243 |  |
| Coccinellinae | Sticholotini | *Sticholotis* sp. | SRR11576242 |  |
| Coccinellinae | Chilocorini | *Chilocorus bijugus* | SRR11576241 |  |
| Coccinellinae | Aspidimerini | *Cryptogonus orbiculus* | SRR11576240 |  |
| Coccinellinae | Aspidimerini | *Cryptogonus hainanensis* | SRR11576239 |  |
| Coccinellinae | Platynaspini | *Platynaspis maculosa* | SRR11576238 |  |
| Coccinellinae | Platynaspini | *Platynaspis hainanensis* | SRR11576237 |  |
| Coccinellinae | Scymnini | *Cryptolaemus montrouzieri* | SRR2971116 |  |
| Coccinellinae | Epilachnini | *Henosepilachna vigintioctopunctata* | SRR11576235 |  |
| Coccinellinae | Epilachnini | *Afissula bisquadripunctata* | SRR11576234 |  |
| Coccinellinae | Epilachnini | *Epilachna insignis* | SRR11576233 |  |
| Coccinellinae | Epilachnini | Epilachnini sp. | SRR11576232 |  |
| Coccinellinae | Scymnini | *Scymnus* sp3 | SRR11576231 |  |
| Coccinellinae | Scymnini | *Scymnus* sp2 | SRR11576230 |  |
| Coccinellinae | Scymnini | *Scymnus* sp4 | SRR11576229 |  |
| Coccinellinae | Scymnini | *Scymnus* sp1 | SRR11576228 |  |
| Coccinellinae | Scymnini | *Scymnus* sp5 | SRR11576227 |  |
| Coccinellinae | Scymnini | *Scymnus* sp6 | SRR11576226 |  |
| Coccinellinae | Scymnini | *Pseudoscymnus* sp. | SRR11576224 |  |
| Coccinellinae | Noviini | *Rodolia pumila* | SRR11576223 |  |
| Microweiseinae | Serangiini | *Serangium japonicum* | SRR11576222 |  |
| Microweiseinae | Serangiini | *Delphastus catalinae* | SRR11576221 |  |
| Coleoptera outgroups | | | | |
| Suborder | Superfamily | Species | Data source |  |
| Polyphaga | Coccinelloidea | *Dastarcus helophoroides* | SRR1201401 |  |
| Polyphaga | Curculionoidae | *Cylas brunneus* | SRR3397644 |  |
| Polyphaga | Curculionoidea | *Dendroctonus ponderosae* | OrthoDB 10 |  |
| Polyphaga | Chrysomeloidae | *Anoplophora glabripennis* | OrthoDB 10 |  |
| Polyphaga | Chrysomeloidae | *Leptinotarsa decemlineata* | OrthoDB 10 |  |
| Polyphaga | Cucujoidea | *Aethina tumida* | OrthoDB 10 |  |
| Polyphaga | Cucujoidea | *Cryptolestes ferrugineus* | ERR2260793 |  |
| Polyphaga | Tenebrionoidea | *Tribolium castaneum* | OrthoDB 10 |  |
| Polyphaga | Tenebrionoidea | *Tenebrio molitor* | SRR1291244 |  |
| Polyphaga | Tenebrionoidea | *Mylabris cichorii* | SRR1996329 |  |
| Polyphaga | Bostrichoidea | *Stegobium paniceum* | SRR1963786 |  |
| Polyphaga | Scarabaeoidea | *Oryctes borbonicus* | OrthoDB 10 |  |
| Polyphaga | Scarabaeoidea | *Allomyrina dichotoma* | SRR5837491 |  |
| Polyphaga | Scarabaeoidea | *Onthophagus taurus* | OrthoDB 10 |  |
| Polyphaga | Staphylinoidea | *Nicrophorus vespilloides* | OrthoDB 10 |  |
| Polyphaga | Elateroidea | *Melanotus cribricollis* | SRR6286841 |  |
| Polyphaga | Elateroidea | *Pyrocoelia pectoralis* | SRR5936558 |  |
| Polyphaga | Buprestoidea | *Agrilus planipennis* | OrthoDB 10 |  |
| Adephaga | Dytiscoidea | *Liopterus haemorrhoidalis* | SRR5892098 |  |
| Adephaga | Dytiscoidea | *Amphizoa lecontei* | SRR5892093 |  |
| Adephaga | Caraboidea | *Carabus granulatus* | SRR596983 |  |
| Archostemata | Cupedoidea | *Priacma serrata* | SRR596769 |  |

**Table S4** Information of the putative eukaryotic *cwh* genes detected from NCBI whole genome shotgun (WGS) assemblies. The protein sequence of *cwh* gene of *Cryptolaemus montrouzieri* (CM_C1a) was used as query to search against the dataset of NCBI whole genome shotgun (WGS) assemblies using tBLASTn. Eukaryotic hits with E-values lower than 10^-10^ and coverage >90% are considered as *cwh* homologs.

| group | species | region | evalue | identity |
| --- | --- | --- | --- | --- |
| Nematoda | *Acrobeloides nanus* | OZJO01012225.1:7645-7223 | 1.76E-29 | 44% |
| Nematoda | *Acrobeloides nanus* | OZJO01015288.1:16180-15782 | 2.57E-14 | 36% |
| Nematoda | *Caenorhabditis* sp. | UNPJ02017940.1:1427-1804 | 8.81E-17 | 37% |
| Nematoda | *Caenorhabditis* sp. | UNPJ02024008.1:598-975 | 8.89E-18 | 40% |
| Nematoda | *Caenorhabditis* sp. | UNPJ02025204.1:713-1090 | 2.88E-15 | 38% |
| Nematoda | *Caenorhabditis* sp. | UNPJ02025949.1:5822-5445 | 2.03E-17 | 40% |
| Nematoda | *Caenorhabditis* sp. | UNPJ02025949.1:6873-7250 | 1.03E-14 | 38% |
| Nematoda | *Caenorhabditis* sp. | UNPJ02027279.1:3243-2866 | 2.00E-16 | 39% |
| Nematoda | *Diploscapter coronatus* | BBTA01000340.1:29321-29701 | 9.06E-40 | 56% |
| Nematoda | *Diploscapter coronatus* | BBTA01000392.1:318628-319011 | 5.41E-43 | 60% |
| Nematoda | *Diploscapter pachys* | LIAE01007271.1:50106-50483 | 2.37E-43 | 60% |
| Nematoda | *Diploscapter pachys* | LIAE01010027.1:66919-67302 | 1.71E-51 | 64% |
| Nematoda | *Halicephalobus mephisto* | SWDT01000049.1:519338-519721 | 9.24E-49 | 64% |
| Nematoda | *Halicephalobus mephisto* | SWDT01000152.1:4033-4416 | 1.28E-49 | 65% |
| Nematoda | *Halicephalobus mephisto* | SWDT01000174.1:94091-94474 | 2.64E-49 | 63% |
| Nematoda | *Halicephalobus* sp. | VOSG01000820.1:8528-8911 | 6.21E-51 | 67% |
| Nematoda | *Heterorhabditis bacteriophora* | ACKM01000890.1:94162-93821 | 9.21E-26 | 50% |
| Nematoda | *Mesorhabditis belari* | UZWA01000059.1:689505-689368;  687137-686853 | 2E-17 | 50% |
| Nematoda | *Mesorhabditis belari* | UZWA01000803.1:11788-12078;  12265-12435 | 2.69E-12 | 54% |
| Nematoda | *Mesorhabditis belari* | UZWA01000997.1:11478-11266;  10963-10679 | 1.31E-23 | 54% |
| Nematoda | *Mesorhabditis belari* | UZWA01001084.1:17950-17624;  17433-17266 | 7E-10 | 38% |
| Nematoda | *Mesorhabditis belari* | UZWC01006569.1:19-420 | 6.70E-44 | 56% |
| Nematoda | *Mesorhabditis belari* | UZWC01007018.1:28-423 | 3.57E-24 | 44% |
| Nematoda | *Mesorhabditis belari* | UZWC01022060.1:19-432 | 1.35E-39 | 51% |
| Nematoda | *Mesorhabditis belari* | UZWC01027784.1:1006-1404 | 2.63E-31 | 45% |
| Nematoda | *Micoletzkya japonica* | UENP01006484.1:461-907 | 3.59E-22 | 39% |
| Nematoda | *Oscheius* sp. | LNBV01010190.1:1196-819 | 2.47E-17 | 37% |
| Nematoda | *Oscheius* sp. | LTAH01046216.1:9-335 | 3.67E-15 | 38% |
| Nematoda | *Oscheius tipulae* | FXWY01000010.1:1488323-1488712 | 8.27E-23 | 40% |
| Nematoda | *Oscheius tipulae* | FXWY01000037.1:171565-171284 | 4.61E-11 | 42% |
| Nematoda | *Oscheius tipulae* | FXWY01000037.1:175255-174812 | 1.38E-17 | 39% |
| Nematoda | *Oscheius tipulae* | FXWY01000037.1:175795-176181 | 1.39E-28 | 48% |
| Nematoda | *Oscheius tipulae* | FXWY01000047.1:60530-60928 | 1.83E-26 | 46% |
| Nematoda | *Panagrolaimus davidi* | CABFKP010008280.1:849-1226 | 4.57E-34 | 50% |
| Nematoda | *Panagrolaimus davidi* | CABFKP010015500.1:1143-1523 | 3.37E-44 | 63% |
| Nematoda | *Panagrolaimus davidi* | CABFKP010022085.1:889-512 | 3.99E-29 | 47% |
| Nematoda | *Panagrolaimus* sp. | CABFKM010001479.1:34-420 | 7.46E-50 | 61% |
| Nematoda | *Panagrolaimus* sp. | CABFKM010003694.1:392-45 | 2.86E-23 | 44% |
| Nematoda | *Panagrolaimus* sp. | CABFKM010008607.1:7328-6948 | 2.54E-31 | 48% |
| Nematoda | *Panagrolaimus* sp. | CABFKO010020092.1:2258-1941;  1928-1692 | 1.72E-21 | 46% |
| Nematoda | *Panagrolaimus* sp. | CABFKQ010010906.1:368-757 | 4.13E-47 | 60% |
| Nematoda | *Panagrolaimus* sp. | CABFKQ010017335.1:453-79 | 1.08E-27 | 43% |
| Nematoda | *Plectus murrayi* | LZQM01012031.1:453-836 | 2.51E-47 | 59% |
| Nematoda | *Plectus murrayi* | LZQM01021385.1:2656-3039 | 6.81E-49 | 63% |
| Nematoda | *Plectus murrayi* | LZQM01032739.1:171-557 | 2.40E-50 | 60% |
| Nematoda | *Plectus sambesii* | NIUQ01000412.1:48420-48803 | 8.71E-36 | 52% |
| Nematoda | *Plectus sambesii* | NIUQ01002487.1:1108-1488 | 5.26E-41 | 58% |
| Nematoda | *Plectus sambesii* | NIUQ01002839.1:7192-7560 | 2.55E-44 | 63% |
| Nematoda | *Plectus sambesii* | NIUQ01003208.1:9403-9789 | 4.23E-46 | 62% |
| Nematoda | *Plectus sambesii* | NIUQ01005329.1:2730-3110 | 3.59E-42 | 59% |
| Nematoda | *Plectus sambesii* | NIUQ01006967.1:5961-6347 | 4.16E-46 | 62% |
| Nematoda | *Rhabditida* sp. | SCGM01000909.1:6842-6468 | 2.76E-23 | 44% |
| Nematoda | *Rhabditida* sp. | SCGM01000909.1:9915-10286 | 7.50E-32 | 52% |
| Nematoda | *Rhabditida* sp. | SCGM01002358.1:5142-4768 | 5.46E-31 | 49% |
| Nematoda | *Rhabditida* sp. | SCGM01003026.1:4771-4526;  4472-4359 | 8.56E-24 | 55% |
| Nematoda | *Rhabditida* sp. | SCGM01004149.1:2903-2538 | 1.13E-20 | 38% |
| Nematoda | *Rhabditida* sp. | SCGM01006017.1:1929-2294 | 2.88E-20 | 37% |
| Tardigrada | *Hypsibius dujardini* | LMYF01000532.1:23224-22835 | 2.05E-28 | 46% |
| Tardigrada | *Hypsibius dujardini* | LMYF01000669.1:10029-9640 | 1.33E-29 | 46% |
| Tardigrada | *Hypsibius dujardini* | LMYF01006863.1:5672-6079 | 1.25E-29 | 46% |
| Tardigrada | *Hypsibius dujardini* | LRSR01000218.1:10029-9640 | 1.33E-29 | 46% |
| Tardigrada | *Hypsibius dujardini* | LRSR01000757.1:23224-22835 | 2.05E-28 | 46% |
| Tardigrada | *Hypsibius dujardini* | LRSR01004763.1:5672-6079 | 1.25E-29 | 46% |
| Tardigrada | *Hypsibius dujardini* | MTYJ01000037.1:494610-494999 | 2.06E-28 | 46% |
| Tardigrada | *Hypsibius dujardini* | MTYJ01000235.1:3940-4347 | 1.26E-29 | 46% |
| Tardigrada | *Ramazzottius varieornatus* | BDGG01000019.1:345935-346372 | 4.04E-20 | 38% |
| Chelicerata | *Hypochthonius rufulus* | LBFL01041407.1:3481-3101 | 6.29E-48 | 65% |
| Chelicerata | *Hypochthonius rufulus* | LBFL01042719.1:369-1 | 7.36E-46 | 61% |
| Chelicerata | *Oppiella nova* | CAJFDS010004099.1:1621-1998 | 1.29E-15 | 38% |
| Chelicerata | *Oppiella nova* | CAJFDS010004099.1:6277-5885 | 2.94E-30 | 44% |
| Chelicerata | *Oppiella nova* | CAJFDS010004099.1:7870-7487 | 1.22E-17 | 40% |
| Chelicerata | *Oppiella nova* | CAJFDS010004099.1:9778-9401 | 2.15E-14 | 38% |
| Chelicerata | *Oppiella nova* | CAJFDS010022624.1:1604-1981 | 5.62E-16 | 37% |
| Chelicerata | *Platynothrus peltifer* | LBFO01036928.1:365-3 | 7.76E-48 | 61% |
| Branchiopoda | *Daphnia carinata* | WJBH01000006.1:1668180-1667812 | 6.99E-37 | 54% |
| Branchiopoda | *Daphnia carinata* | WJBH01000006.1:443250-442870 | 5.22E-39 | 57% |
| Branchiopoda | *Daphnia dubia* | JAAVJA010001593.1:19200-18814 | 9.8E-36 | 51% |
| Branchiopoda | *Daphnia dubia* | JAAVJA010012811.1:718-1104 | 2.37E-37 | 52% |
| Branchiopoda | *Daphnia magna* | LRGB01000725.1:784325-784498;  784540-784713 | 1.19E-27 | 55% |
| Branchiopoda | *Daphnia magna* | LRGB01022416.1:4-333 | 2.13E-20 | 45% |
| Branchiopoda | *Daphnia magna* | QYSF01000007.1:2565594-2565421;  2565379-2565206 | 1.38E-27 | 55% |
| Branchiopoda | *Daphnia magna* | QYSF01000007.1:2571318-2570950 | 1.19E-34 | 54% |
| Branchiopoda | *Daphnia magna* | QYSF01000008.1:8848551-8848937 | 2.33E-23 | 45% |
| Branchiopoda | *Daphnia magna* | QYSF01000008.1:8857018-8857401 | 1.41E-26 | 45% |
| Branchiopoda | *Daphnia magna* | QYSF01000032.1:5160-4774 | 5.12E-25 | 45% |
| Branchiopoda | *Daphnia pulex* | ACJG01000252.1:11845-12228 | 1.34E-33 | 50% |
| Branchiopoda | *Daphnia pulex* | ACJG01000252.1:13254-13640 | 1.46E-36 | 52% |
| Branchiopoda | *Daphnia pulex* | ACJG01001303.1:4166-4297;  4405-4533 | 6.39E-17 | 50% |
| Branchiopoda | *Daphnia pulex* | FLTH02000006.1:2850045-2849659 | 1.49E-34 | 50% |
| Branchiopoda | *Daphnia pulex* | FLTH02000006.1:2851664-2851278 | 4.31E-33 | 48% |
| Branchiopoda | *Daphnia pulex* | FLTH02000083.1:285275-284904 | 6.89E-13 | 38% |
| Branchiopoda | *Daphnia pulex* | FLTH02000356.1:19570-19974 | 5.38E-17 | 39% |
| Branchiopoda | *Eulimnadia texana* | NKDA01000002.1:8949492-8949082 | 7.7E-37 | 50% |
| Branchiopoda | *Eulimnadia texana* | NKDA01000002.1:8957137-8956751 | 1.75E-44 | 58% |
| Branchiopoda | *Eulimnadia texana* | NKDA01000002.1:8960861-8960472 | 7.07E-33 | 49% |
| Branchiopoda | *Eulimnadia texana* | NKDA01000002.1:9659598-9659449;  9659068-9658826 | 2E-12 | 59% |
| Branchiopoda | *Eulimnadia texana* | NKDA01000004.1:8849217-8848831 | 7.89E-46 | 58% |
| Branchiopoda | *Eulimnadia texana* | NKDA01000004.1:8851632-8851411;  8851052-8850819 | 8E-47 | 42% |
| Collembola | *Ceratophysella communis* | VNWX01024910.1:717-340 | 5.07E-49 | 61% |
| Collembola | *Folsomia candida* | LNIX01000014.1:470241-470654 | 1.84E-29 | 48% |
| Collembola | *Folsomia candida* | LNIX01000038.1:617278-616907 | 4.00E-35 | 54% |
| Collembola | *Folsomia candida* | LNIX01000038.1:625216-625079;  625070-624837 | 6.27E-25 | 70% |
| Collembola | *Folsomia candida* | LNIX01000038.1:646084-645704 | 3.73E-35 | 52% |
| Collembola | *Mesaphorura yosii* | WIXG01000449.1:43507-43923 | 2.77E-23 | 42% |
| Collembola | *Neelides* sp. | VJMY01002578.1:2567-2421;  2284-2063 | 9.66E-15 | 49% |
| Collembola | *Orchesella cincta* | LJIJ01000381.1:109294-109653 | 1.65E-24 | 47% |
| Collembola | *Pseudachorutes palmiensis* | VNWY01002632.1:3255-3644 | 9.72E-49 | 63% |
| Collembola | *Pseudachorutes palmiensis* | VNWY01002632.1:4853-5242 | 1.02E-47 | 62% |
| Collembola | *Pygmarrhopalites habei* | WJFB01012424.1:1008-1388 | 1.49E-42 | 57% |
| Collembola | *Pygmarrhopalites habei* | WJFB01020784.1:483-863 | 1.51E-28 | 47% |
| Collembola | *Sinella curviseta* | RBVU02000001.1:64318940-64318560 | 1.41E-28 | 47% |
| Collembola | *Sinella curviseta* | RBVU02000005.1:45453492-45453103 | 4.27E-30 | 48% |
| Collembola | *Tomocerus minor* | JAAAYG010001724.1:6732-7112 | 1.12E-49 | 65% |
| Collembola | *Tomocerus minor* | JAAAYG010006526.1:6505-6885 | 3.55E-46 | 61% |
| Collembola | *Tomocerus minor* | JAAAYG010007778.1:46897943-46898323 | 6.12E-41 | 55% |
| Collembola | *Tomocerus minor* | JAAAYG010007778.1:47183510-47183890 | 1.74E-42 | 56% |
| Insecta | *Bradysia coprophila* | VSDI01000186.1:1934289-1934675 | 4.00E-44 | 60% |
| Insecta | *Bradysia coprophila* | VSDI01000706.1:6287186-6286830 | 2.00E-29 | 50% |
| Insecta | *Contarinia nasturtii* | VYII01002452.1:c130149-129787 | 3.00E-36 | 56% |
| Insecta | *Culicoides sonorensis* | OGVF02000315.1:163261-163698 | 9.00E-23 | 39% |
| Insecta | *Culicoides sonorensis* | OGVF02000581.1:c1387-1004 | 5.00E-35 | 51% |
| Insecta | *Culicoides sonorensis* | OGVF02001256.1:53322-53702 | 7.00E-44 | 59% |
| Insecta | *Culicoides sonorensis* | OGVF02001256.1:53964-54344 | 3.00E-16 | 40% |
| Insecta | *Culicoides sonorensis* | OGVF02004821.1:648-1073 | 1.00E-27 | 44% |
| Insecta | *Gerris buenoi* | JHBY02094181.1:710-820;  JHBY02094182.1:1598-1861 | 4.00E-23 | 57% |

**Table S5** Selection pressures of *cwh1* and *cwh2* across Coccinellinae calculated by SLAC in HYPER software (*p* value threshold of 0.05).

|  | No. of sequences | dN/dS | Total sites | Negative | Positive |
| --- | --- | --- | --- | --- | --- |
| *cwh1* | 43 | 0.144 | 162 | 97 | 1 |
| *cwh2* | 41 | 0.159 | 199 | 106 | 0 |

**Table S6** Primers for ladybird *cwh* used for cloning. Sequences are given for the forward (F) and reverse (R) primers and the annealing temperature used for PCR (*T*m) with each primer pair.

| Species | Gene ID | F/R | Primer sequence (5’ - 3’) | *T*m |
| --- | --- | --- | --- | --- |
| *Cryptolaemus*  *montrouzieri* | CM-C1a | F | ATGAGTGACAGAGAAACTTTCGCT | 60 |
|  |  | R | TCATTGTTTGGCCTTGTAAAACACATGATC |  |
| *Harmonia*  *axyridis* | HA-C1b | F | ATGAGTGACCGAGATATTTTTGCCA | 60 |
|  |  | R | TCATTTTTCTTTGTAGAATACATGATTGCCAAC |  |

**Table S7** Primers for constructing *cwh*-RNAi strains of *Cryptolaemus montrouzieri*. Promoter sequences of T7 RNA polymerase are bolded.

| Target gene | Primer name | Primer sequence (5’ - 3’) |
| --- | --- | --- |
| CM-C1a | CM-C1aF | GAAGCTAGAGGCGAACCTGA |
|  | CM-C1aR | CGGAAGAAAGAGGAGCATCA |
|  | CM-C1aT7F | **GGATCCTAATACGACTCACTATAGG**GAAGCTAGAGGCGAACCTGA |
|  | CM-C1aT7R | **GGATCCTAATACGACTCACTATAGG**CGGAAGAAAGAGGAGCATCA |
| CM-C1b | CM-C1bF | GGATGGCAGAGAAGTTTTCG |
|  | CM-C1bR | GCTCGGGTTGTTGTAGTGGT |
|  | CM-C1bT7F | **GGATCCTAATACGACTCACTATAGG**GGATGGCAGAGAAGTTTTCG |
|  | CM-C1bT7R | **GGATCCTAATACGACTCACTATAGG**GCTCGGGTTGTTGTAGTGGT |
| CM-C2 | CM-C2F | CCCGGTGCAACTGTTCTAAT |
|  | CM-C2R | AGAACTTCCGGTGGGATCTT |
|  | CM-C2T7F | **GGATCCTAATACGACTCACTATAGG**CCCGGTGCAACTGTTCTAAT |
|  | CM-C2T7R | **GGATCCTAATACGACTCACTATAGG**AGAACTTCCGGTGGGATCTT |
| Control | GFPF | AAGGGCGAGGAGCTGTTCACCG |
|  | GFPR | CAGCAGGACCATGTGATCGCGC |
|  | GFPT7F | **GGATCCTAATACGACTCACTATAGG**AAGGGCGAGGAGCTGTTCACCG |
|  | GFPT7R | **GGATCCTAATACGACTCACTATAGG**CAGCAGGACCATGTGATCGCGC |

**Table S8** Information of fossils used for calibration points in the species phylogenetic analysis. MRCA: most recent common ancestor.

| No. | Calibrated node | Note | Fossil taxa | Min. age | Reference |
| --- | --- | --- | --- | --- | --- |
| 1 | Crown Coccinellini | *Coccinella sodoma* | Coccinellidae - Coccinellini | 33.9 | [84] |
| 2 | Crown Serangiini | *Serangium kalandyki* | Coccinellidae - Serangiini | 33.9 | [85] |
| 3 | Crown Coccinellidae | *Rhyzobius antiquus* | Coccinellidae - Coccidulini | 48.6 | [86] |
| 4 | Crown Coccinelloidea | *Archelatrius marinae* | Coccinelloidea- Latridiidae | 125.5 | [87] |
| 5 | Crown Chrysomeloidea | *Cretoprionus liutiaogouensis* | Chrysomeloidea - Cerambycidae | 122.5 | [88] |
| 6 | Crown Curculionoidea | *Nanophydes ovatus* | Curculionoidea - Brentidae | 155.7 | [89] |
| 7 | Crown Cucujoidea | *Nitidulina eclavata* | Cucujoidea - Nitidulidae | 155.7 | [90] |
| 8 | Crown Tenebrionoidea | *Jurallecula grossa* | Tenebrionoidea - Tenebrionidae | 155.7 | [91] |
| 9 | MRCA of Bostrichoidea  + Cucujiformia | *Paradermestes jurassicus* | Bostrichoidea - Dermestidae | 155.7 | [92] |
| 10 | MRCA of  Staphylinoidea +  Scarabaeoidea | *Juraesalus atavus* | Scarabaeoidea - Lucanidae | 155.7 | [93] |
| 11 | MRCA of Buprestoidea + Elateroidea | *Ancestrimorpha volgensis* | Buprestoidea - Buprestidae | 164.7 | [94] |
| 12 | Crown Adephaga | *Sogdodromeus altus* | Caraboidea -  Trachypachidae | 221.5 | [95] |
